# ZBTB7B inhibits glioma tumorigenicity by upregulating GPR17 and CXCL10

**DOI:** 10.1093/jmcb/mjaf043

**Published:** 2025-11-26

**Authors:** Linmei Zhang, Haozhe Zhang, Chenxi Wang, Aoxin Jiang, Fei Zhao, Sifan Yang, Hong Lei, Xuelan Yu, Juan Ren, Chengfang Tang, Xiaofei Wang, Yanke Chen

**Affiliations:** School of Stomatology, Xi’an Medical University, Xi’an 710021, China; The Second Clinical Medical College, Health Science Center, Xi’an Jiaotong University, Xi’an 710061, China; The Second Clinical Medical College, Health Science Center, Xi’an Jiaotong University, Xi’an 710061, China; The Second Clinical Medical College, Health Science Center, Xi’an Jiaotong University, Xi’an 710061, China; Biobank, Xi’an People’s Hospital (Xi’an Fourth Hospital), Xi’an 710004, China; Department of Cell Biology and Genetics, School of Basic Medical Sciences, Xi’an Jiaotong University, Xi’an 710061, China; Institute of Basic and Translational Medicine, Xi’an Medical University, Xi’an 710021, China; Department of Cell Biology and Genetics, School of Basic Medical Sciences, Xi’an Jiaotong University, Xi’an 710061, China; Department of Oncology Radiotherapy, First Affiliated Hospital of Xi’an Jiaotong University, Xi’an 710061, China; School of Stomatology, Xi’an Medical University, Xi’an 710021, China; Biomedical Experimental Center, Xi’an Jiaotong University, Xi’an 710061, China; Department of Cell Biology and Genetics, School of Basic Medical Sciences, Xi’an Jiaotong University, Xi’an 710061, China; Institute of Genetics and Developmental Biology, Xi’an Jiaotong University, Xi’an 710061, China; Key Laboratory of Environment and Genes Related to Diseases, Xi’an Jiaotong University, Xi’an 710061, China; Hebei Key Laboratory of Colorectal Cancer Precision Diagnosis and Treatment, The First Hospital of Hebei Medical University, Shijiazhuang 050000, China

**Keywords:** glioma, ZBTB7B, GPR17, CXCL10, tumorigenicity

## Abstract

The transcription factor ZBTB7B has been identified as a potential tumor suppressor through a CRISPR–Cas9-based functional screen of tumor-associated genes, as overexpression of ZBTB7B could significantly suppress tumor growth in the models of breast cancer brain metastasis, which prompted our further exploration of its inhibitory role in glioma. To elucidate the underlying mechanisms of this suppressive effect, lentiviral-mediated ZBTB7B overexpression was established in U118 and GL261 glioma cell lines, and systematic evaluation of tumorigenic capacity was performed through *in vitro* and xenograft assays. The results showed that ZBTB7B transcriptionally activated GPR17 expression, which suppressed protein kinase A phosphorylation, amplified mitochondrial reactive oxygen species generation, and triggered Caspase3-dependent apoptosis. Meanwhile, ZBTB7B upregulated CXCL10 secretion, which markedly enhanced CD4^+^ and CD8^+^ T cell accumulation. Clinical validation through multiplex immunofluorescence staining on a tissue microarray of 129 glioma samples revealed a progressive loss of ZBTB7B protein expression across WHO grades II to IV, inversely correlating with tumor malignancy. These findings demonstrate ZBTB7B as a dual-function tumor suppressor that concurrently induces intrinsic apoptosis and remodels the tumor immune microenvironment in glioma toward a ‘hot’ phenotype. Therefore, we propose ZBTB7B reactivation as a novel therapeutic strategy for glioma.

## Introduction

Glioma, the most common primary tumor of the central nervous system, is classified into four grades according to its cellular morphology, proliferation rate, and aggressiveness (Louis et al., 2007; Modrek et al., 2014; Perry and Wesseling, 2016; Darlix et al., 2017; Bikfalvi et al., 2023). Standard treatment of the disease generally involves a multimodal approach, combining surgical resection, radiotherapy, and chemotherapy (Chen et al., 2017a; Nabors et al., 2020). Despite these interventions, patient outcomes remain poor, with a median survival of only 15–16 months and a 10-year survival rate of 0.71% in adults (Tamimi and Juweid, 2017; Tykocki and Eltayeb, 2018). Improving the diagnosis and treatment of this aggressive malignancy requires the identification of key molecular regulators that drive its pathogenesis (Van Meir et al., 2010; Hu et al., 2018; Zuccarini et al., 2018; Khabibov et al., 2022). Despite the accumulated knowledge of this disease, the molecular mechanisms underlying its progression are not fully elucidated.

In our previous study, a CRISPR-based screen in MDA-MB-231-Br-HER2 (231BR) cells identified the transcription factor zinc finger and BTB domain containing 7B (ZBTB7B) as a tumor suppressor that significantly inhibited cell proliferation in breast cancer brain metastasis (Chen et al., 2017b, 2019, 2020). ZBTB7B (also known as ThPOK) is a member of the ZBTB transcription factor family (Lee and Maeda, 2012). It regulates gene expression by promoting target gene transcription and recruiting chromatin remodeling complexes (e.g. deacetylases) to suppress transcription where necessary (Albagli et al., 1995; Chang et al., 1996; Ahmad et al., 2003). Studies have demonstrated that ZBTB7B plays a pivotal role in thymocyte differentiation by directing precursor cells toward the CD4^+^ T cell lineage in the thymus (He et al., 2005, 2008, 2010; Naito and Taniuchi, 2010). Recent research has expanded its known functions to include regulation of other T cell subpopulations, such as natural killer (NK) T cell activation (Park et al., 2010; Kappes, 2010; Chakravarti and Godfrey, 2010; Engel et al., 2012; Basu et al., 2023). Given its established role in T cell differentiation and immune differentiation, ZBTB7B has emerged as a potential key player in tumor immunotherapy (Wang et al., 2024). However, its function in glioma remains unexplored.

Genomic analyses of data from the Cancer Genome Atlas (TCGA) database have identified missense mutations in the ZBTB7B gene across multiple tumor types. For instance, in bladder cancer, these mutations are linked to tumor progression (Heide et al., 2019; Kennedy et al., 2020; Liang et al., 2020; Cui et al., 2022; Zhu et al., 2024). This evidence implies that ZBTB7B dysfunction may promote tumorigenesis. Nevertheless, direct experimental evidence for the tumor-suppressive effect of ZBTB7B in glioma is scarce. To address this gap, the present study investigates the potential role of ZBTB7B in glioma and its underlying molecular mechanisms.

## Results

### ZBTB7B inhibits proliferation and induces apoptosis of glioma cells *in vitro*

**Figure 1:**
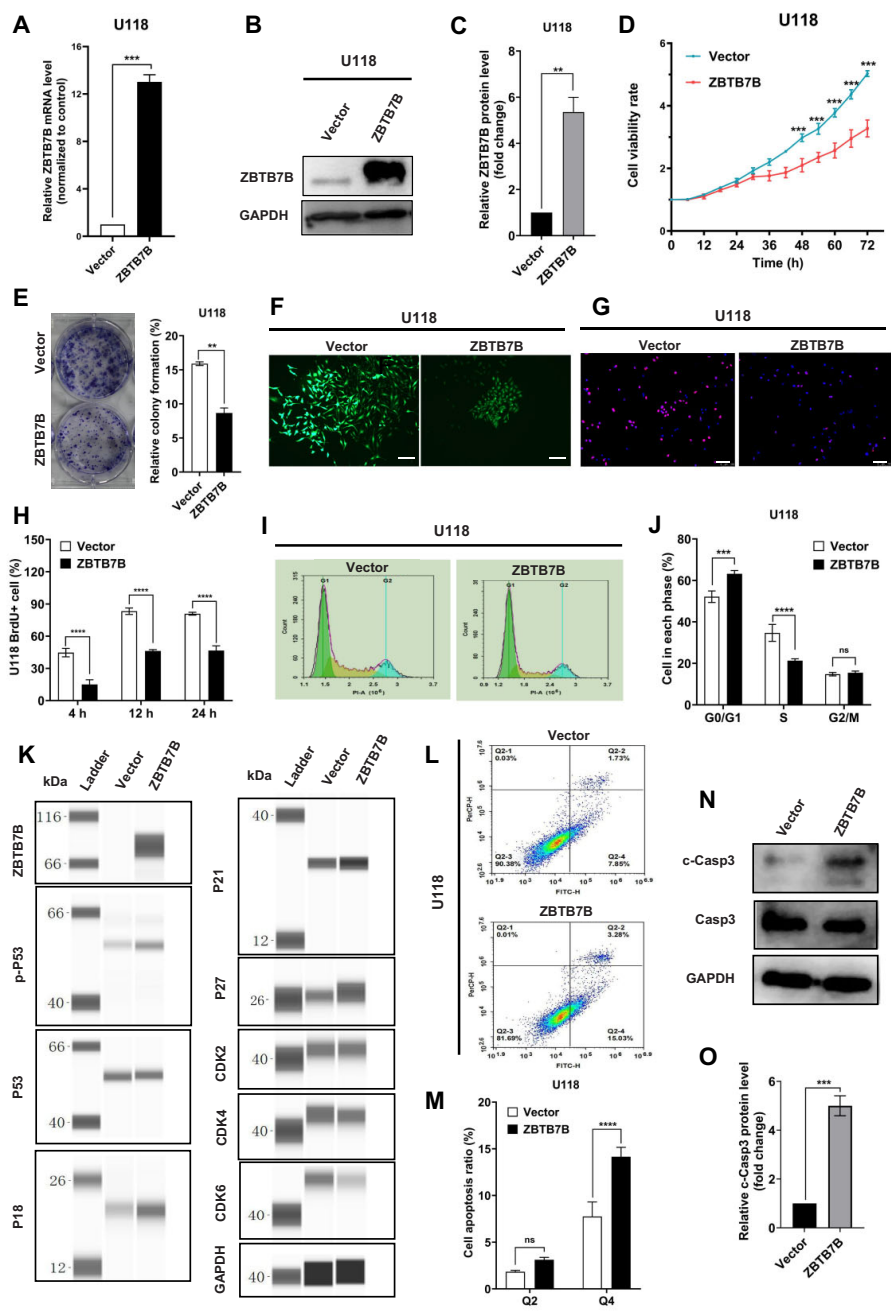
Overexpression of ZBTB7B in human glioma U118 cells inhibits cell growth *in vitro*, arrestes cell cycle, and induces apoptosis. (**A**–**C**) qRT-PCR and western blot analyses of ZBTB7B expression in U118 cells transduced with control vector or lentiviral vector for ZBTB7B overexpression. *n* = 3 biologically independent experiments. (**D**) Real-time monitoring of cell viability by using a live-cell imaging system. Data were normalized and statistically analyzed between ZBTB7B-overexpressing and control cells. (**E**) 2D clonogenic assays evaluating U118 cell clonogenic potential by quantitative analysis of the colony formation rate (%). (**F**) 3D clonogenic assays demonstrating U118 cell growth in 3D culture. Scale bar, 250 μm. (**G** and **H**) BrdU incorporation assays evaluating U118 cell proliferation by quantifying the proportion of BrdU-positive cells in the total cell population. Scale bar, 100 μm. (**I** and **J**) Flow cytometry analysis of cell cycle alterations in U118 cells after stable ZBTB7B overexpression. (**K**) WES analysis of protein expression levels related to cell cycle regulation, including p-P53, P53, P21, P27, P18, CDK2, CDK4, and CDK6. GAPDH was used as an internal reference. (**L** and **M**) Flow cytometry analysis of U118 cell apoptosis. (**N** and **O**) Western blot analysis of c-Casp3 protein level, quantified with triplicate repetition. All experiments were conducted independently three times. **P < 0.01, ***P < 0.001, ****P < 0.0001; ns, not significant.

*In vitro* experiments were conducted to elucidate the potential tumor-suppressive role of ZBTB7B in glioma. The human glioma cell line U118 and the murine glioma cell line GL261 were genetically modified via lentiviral infection to overexpress ZBTB7B (hereafter referred to as U118-ZBTB7B and GL261-ZBTB7B, respectively). Successful ZBTB7B overexpression at both mRNA and protein levels was confirmed through quantitative real-time polymerase chain reaction (qRT-PCR) and western blotting (Figure 1A–C; Supplementary Figure S1A–C).

Dynamic changes in cell number were monitored over a 72-h period using a live-cell imaging workstation. ZBTB7B-overexpressing cells exhibited a marked reduction in proliferation compared with their vector control counterparts (Figure 1D; Supplementary Figure S1D). Both two-dimensional (2D) and three-dimensional (3D) clonogenic assays revealed that ZBTB7B overexpression significantly suppressed the colony-forming capacity of U118 and GL261 cells, reducing both the density and size of cell colonies (Figure 1E and F; Supplementary Figure S1E and F). In addition, BrdU incorporation assays showed that ZBTB7B overexpression significantly reduced BrdU incorporation in tumor cells, signifying a potent inhibitory effect on DNA synthesis and cell proliferation (Figure 1G and H; Supplementary Figure S1G and H).

The effect of ZBTB7B overexpression on glioma cell cycle was analyzed by propidium iodide (PI) staining. In both U118 and GL261 glioma cells, elevated ZBTB7B expression markedly increased the proportion of cells in the G1 phase while substantially reducing the proportion in the S phase (Figure 1I and J; Supplementary Figure S1I and J). Digital western (WES) analysis revealed that ZBTB7B overexpression enhanced the phosphorylation of P53 (a critical cell cycle checkpoint regulator) and the expression of P21, P27, and P18 (cell cycle inhibitors) and concurrently decreased the levels of CDK2, CDK4, and CDK6 (cyclin-dependent kinases) (Figure 1K). These results suggested that ZBTB7B-induced cell cycle arrest was mediated through modulation of the P53 signaling pathway and its downstream regulatory proteins.

Annexin V-FITC/PI staining revealed a significant increase in apoptosis in ZBTB7B-overexpressing U118 and GL261 cells (Figure 1L and M; Supplementary Figure S1K and L). Consistent with this, western blot analysis further demonstrated the enhanced cleavage of Caspase3 upon ZBTB7B overexpression (Figure 1N and O).

### ZBTB7B suppresses human glioma U118 tumorigenicity in BALB/c nude mice

U118 glioma cells overexpressing either ZBTB7B or control vector were labeled with green fluorescent protein (GFP)- and luciferase-tagged lentiviral vectors. The luciferase assay confirmed comparable baseline luminescence activities of the two cell groups *in vitro* prior to implantation (Supplementary Figure S2A). An intracranial glioma model was established by injecting the cells into the right lateral ventricle of BALB/c nude mice (Supplementary Figure S2B; McCutcheon et al., 2000). Tumor progression was monitored by bioluminescence imaging on Days 7, 14, and 21 post-inoculation using the IncuCyte Small Animal Imaging System (Supplementary Figure S2C). Tumor growth, indicated by mean fluorescence intensity, was significantly hindered in the ZBTB7B-overexpression group as compared with the control group (Supplementary Figure S2D). On Day 21, brain tissues were extracted after perfusion and paraformaldehyde fixation, and the fluorescent signals from GFP-labeled tumor cells were analyzed. Remarkably, no tumor remnants were detectable in 80% of the mice in the ZBTB7B-overexpression group, highlighting a substantial inhibitory effect on tumor growth *in vivo* (Supplementary Figure S2E).

To examine the temporal dynamics of tumor suppression mediated by ZBTB7B, BALB/c nude mice were inoculated intracranially with control or ZBTB7B-overexpressing glioma cells, and tumor growth was monitored on Days 2, 4, and 6 post-injection. Control mice exhibited progressively increasing GFP-positive tumor signals. In contrast, no GFP-positive signals were detected in the ZBTB7B-overexpression group beyond Day 2 (Supplementary Figure S2F).

Frozen sections of mouse brain were stained with DAPI and analyzed by fluorescence microscopy. Robust GFP signal, indicative of extensive tumor growth, was observed in the control group. In contrast, the ZBTB7B-overexpression group exhibited only minimal GFP signal after inoculation, which gradually diminished to undetectable level (Supplementary Figure S2G).

Hematoxylin and eosin (HE) staining on paraffin-embedded brain sections confirmed the pronounced tumor suppression by ZBTB7B. Immunohistochemical assessment revealed markedly reduced cell proliferation (Ki67^+^ cells) and enhanced apoptosis (TUNEL^+^ cells) in the ZBTB7B-overexpression group (Supplementary Figure S2H).

### ZBTB7B suppresses murine glioma GL261 tumorigenicity in C57BL/6 mice

**Figure 2:**
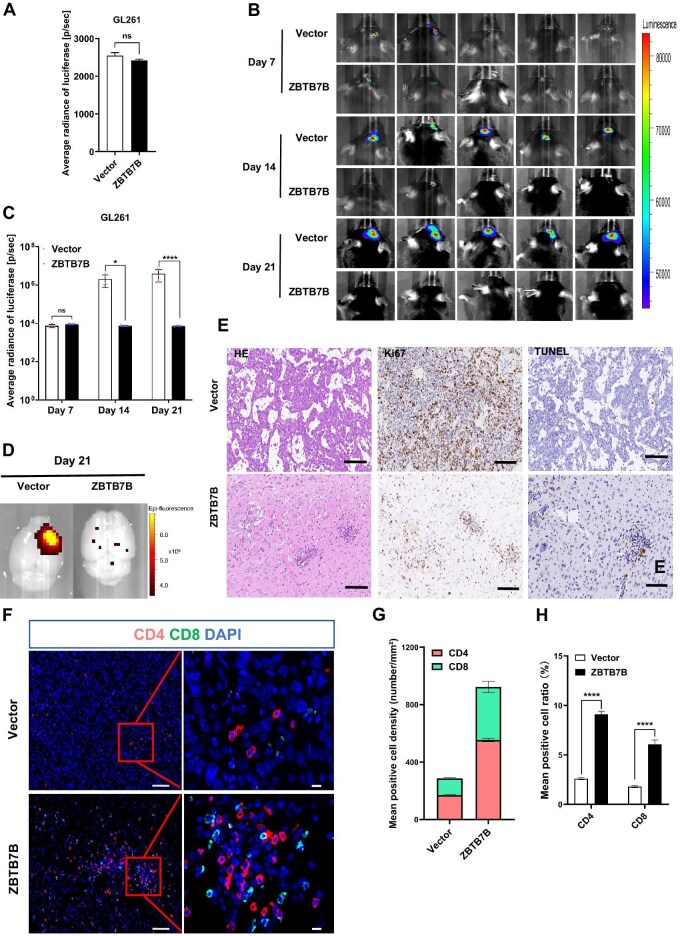
ZBTB7B inhibits glioma tumorigenicity in C57BL/6 mice. (**A**) Evaluation of luciferase activity in GL261 cells cultured *in vitro* (*P* = 0.07). (**B** and **C**) Tumor growth in the brain was monitored on Days 7, 14, and 21 using the IncuCyte^®^ Small Animal *in vivo* Imaging System and the luciferase activity was quanfied. Vector (*n* = 5); ZBTB7B (*n* = 5). (**D**–**H**) Mouse brain tissues were collected on Day 21. (**D**) GFP fluorescence signals of tumor cells in brain tissues. (**E**) The brain tumor tissues were stained with HE, Ki67, and TUNEL. Scale bar, 50 μm. (**F**) Multiplex immunofluorescence staining for DAPI, CD4, and CD8. Scale bar, 50 μm. Partially enlarged view of the boxed area is displayed on the right. Scale bar, 10 μm. (**G**) Densities of CD4^+^ and CD8^+^ cells within tumor regions, calculated as the number of cells per square millimeter (cells/mm^2^). (**H**) Ratios of CD4^+^ and CD8^+^ cells were assessed across groups using three randomly selected fields of equal size. *P < 0.05, ****P < 0.0001; ns, not significant.

The inability of ZBTB7B-overexpressing cells to form neoplasm in nude mice suggested two possibilities. First, the tumor cells might have failed to adapt to the *in vivo* microenvironment, resulting in cell death. Second, the implanted tumor cells might have been targeted and eliminated by the host immune system. To further explore the tumor-suppressive mechanism of ZBTB7B and its relationship with the immune response, tumor transplantation experiments were conducted in the lateral ventricle of wild-type C57BL/6 mice with intact immune system. GL261 glioma cells expressing either ZBTB7B or control vector were labeled with GFP and luciferase. Comparable luciferase activities of the two groups *in vitro* prior to implantation were confirmed (Figure 2A). Bioluminescence imaging on Days 7, 14, and 21 post-implantation revealed a significant suppression of tumor growth in mice implanted with ZBTB7B-overexpressing GL261 cells as compared to the controls (Figure 2B and C). Notably, tumor remnants were undetectable in the ZBTB7B-overexpression group (Figure 2D). Immunohistochemical analysis of brain tissues further demonstrated a marked reduction in tumor cell proliferation (Ki67^+^ cells) and a significant increase in apoptosis (TUNEL^+^ cells) in the ZBTB7B-overexpression group (Figure 2E). These results indicated that ZBTB7B induced both growth arrest and apoptosis in glioma cells.

Interestingly, the residual tumors in the ZBTB7B-overexpression group were surrounded by increased infiltration of CD4^+^ and CD8^+^ T cells (Figure 2F). Quantitative analysis revealed that the mean ratio and density of CD4^+^ and CD8^+^ T cells were significantly higher in tumor specimens from ZBTB7B-overexpressing mice than in those from control mice (Figure 2G and H and Table 1).

**Table 1:** **Analysis of CD4 and CD8 cell positivity via multiplex immunofluorescence staining in ventricular tumor specimens from ZBTB7B-overexpressing and vector control C57BL/6 mice**.

	**Vector**	**ZBTB7B**
**Parameters**	**(mean ± SD)**	**(mean ± SD)**
CD4^+^ cells		
Number	102 ± 4	333 ± 7
Positivity rate (%)	2.61 ± 0.21	9.10 ± 0.54
Positive density (number/mm²)	170.00 ± 6.00	554.33 ± 12.10
CD8^+^ cells		
Number	70 ± 4	222 ± 23
Positivity rate (%)	1.80 ± 0.17	6.08 ± 0.75
Positive density (number/mm²)	116.67 ± 6.03	369.67 ± 38.80

### ZBTB7B regulates G protein-coupled receptor signaling pathways and chemokine secretion

**Figure 3:**
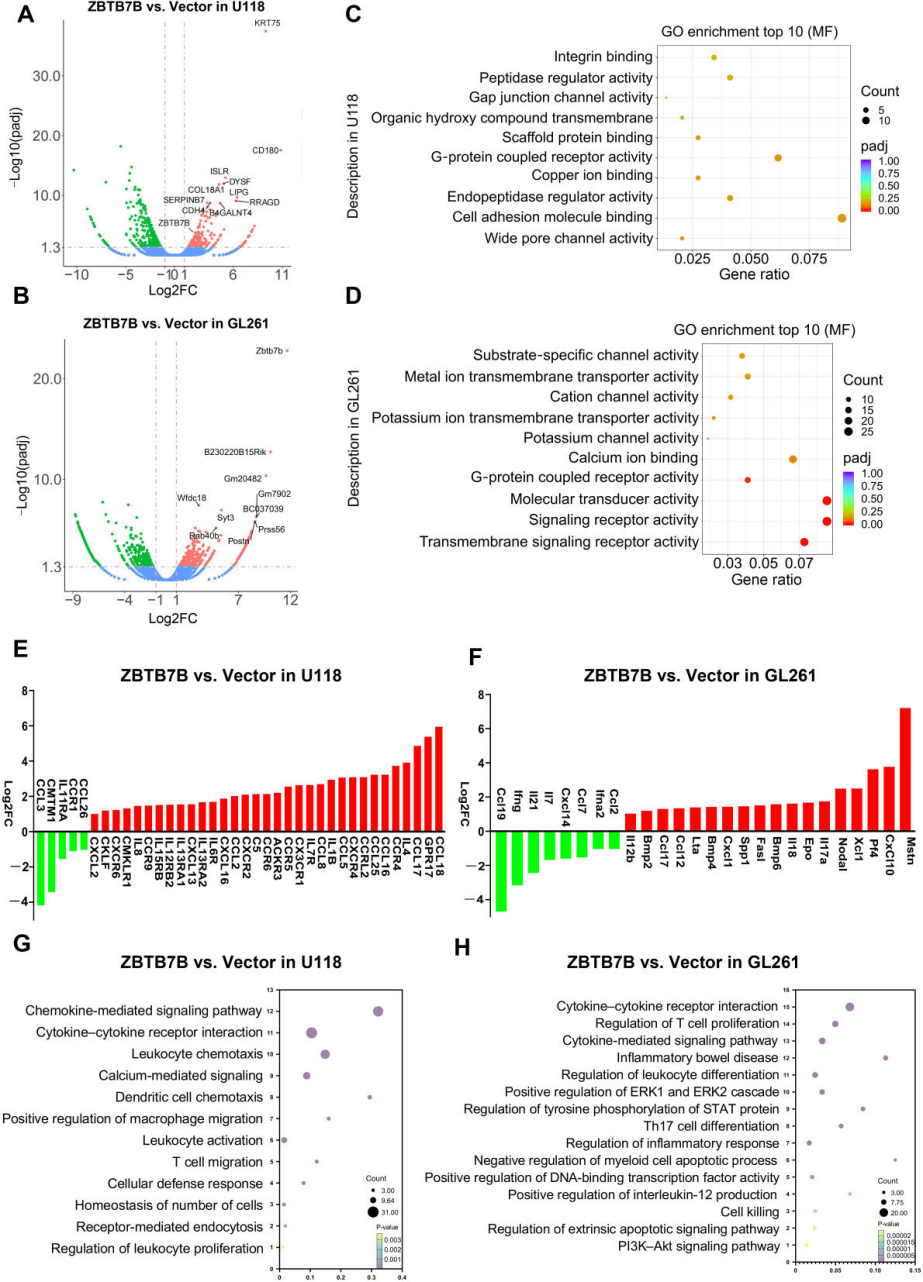
Transcriptome sequencing analysis reveals that ZBTB7B modulates GPCR signaling pathways and influences chemokine secretion. (**A** and **B**) Volcano plots showing DEGs after ZBTB7B overexpression in U118 and GL261 cells. (**C** and **D**) GO enrichment analysis of the upregulated DEGs after ZBTB7B overexpression. (**E** and **F**) qPCR array analysis showing the differentially expressed chemokines and cytokines after ZBTB7B overexpression in U118 and GL261 cells. (**G** and **H**) KEGG pathway enrichment analysis of the upregulated DEGs from qPCR array. Threshold criteria: |log2FC| ≥ 1 and adjusted *P*-value (padj) ≤0.05.

Transcriptome sequencing was performed to identify genes regulated by ZBTB7B. In U118 cells, ZBTB7B overexpression significantly altered the expression of 522 genes (187 upregulated and 335 downregulated) (Figure 3A). Similarly, 1230 differentially expressed genes (DEGs) (577 upregulated and 653 downregulated) were identified in GL261 cells (Figure 3B). Gene ontology (GO) analysis revealed that biological processes related to G protein-coupled receptor (GPCR) activity were enriched in both cell lines (Figure 3C and D). Kyoto Encyclopedia of Genes and Genomes (KEGG) pathway enrichment analysis further identified cytokine–cytokine receptor interaction and cell adhesion molecules as significantly co-enriched pathways (Supplementary Figure S3A and B).

PCR array analysis demonstrated significant upregulation of cytokines and chemokines, notably CCL17, GPR17, and CCL18 in U118 cells (*P* ≤ 0.05, |log2FC| ≥ 3.0; Figure 3E) and Pf4, CXCL10, and Mstn in GL261 cells (*P* ≤ 0.05, |log2FC| ≥ 3.0; Figure 3F). Enrichment analysis of these DEGs further confirmed that ZBTB7B modulated multiple biological processes and pathways, including the regulation of T cell proliferation, and cytokine- and chemokine-mediated signaling pathways (Figure 3G and H). Notably, GPR17 and CXCL10 were identified as hub genes in the enriched pathways, suggesting their critical involvement in ZBTB7B-mediated tumor suppression.

### ZBTB7B inhibits glioma tumorigenicity by regulating GPR17

**Figure 4:**
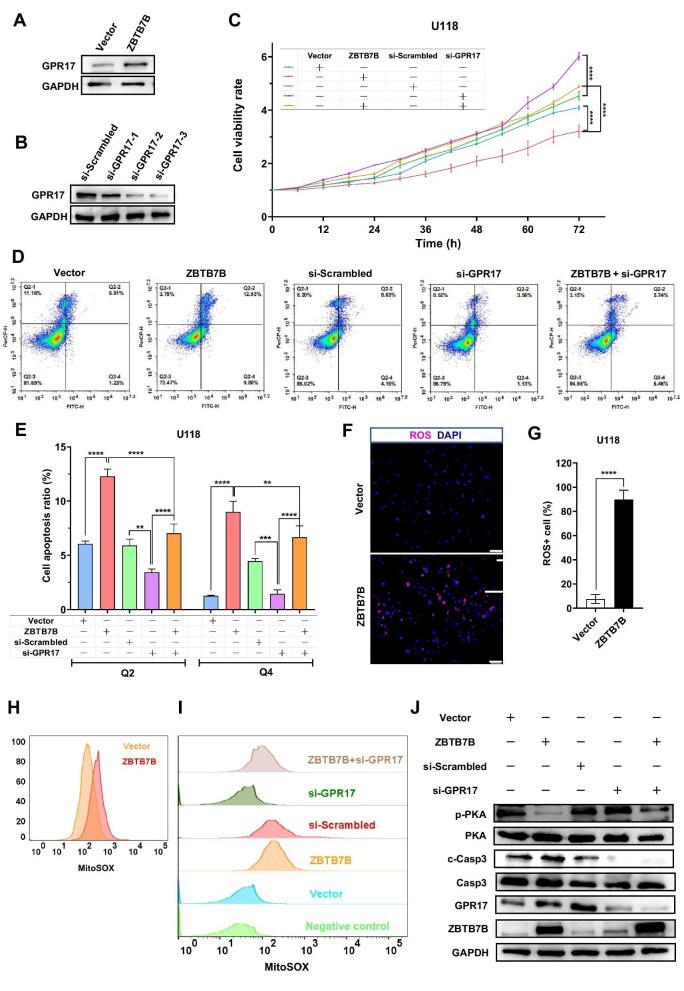
ZBTB7B inhibits glioma tumorigenicity by regulating GPR17 expression. (**A**) Western blot analysis of GPR17 expression in ZBTB7B-overexpressing and control U118 cells. (**B**) Western blot analysis of the efficiency of three siRNAs targeting GPR17 in inhibiting endogenous GPR17 protein expression. (**C**) Real-time monitoring of cell viability by using a live-cell imaging system. Normalized data were statistically analyzed between groups as indicated. (**D** and **E**) Flow cytometry analysis of apoptosis, including early apoptosis and late apoptosis. (**F** and **G**) Detection of mitochondrial ROS levels by using MitoSOX Red (Ex/Em: 369 nm/610 nm). Scale bar, 50 μm. The percentage of ROS^+^ cells was calculated by using Image J and statistically analyzed between groups. (**H** and **I**) Flow cytometry analysis of mitochondrial ROS levels, illustrating the number and fluorescence intensity of positively stained cells. (**J**) Western blot analysis of p-PKA, c-Casp3, ZBTB7B, and GPR17 protein expression. GAPDH was used as an internal reference. All experiments were performed independently at least three times. **P < 0.01, ***P < 0.001, ****P < 0.0001.

GPR17, a member of the GPCR family, has been reported to induce oligodendrocyte apoptosis (Ou et al., 2016). In this study, qRT-PCR analysis showed that ZBTB7B significantly enhanced GPR17 gene transcription (Supplementary Figure S4A). This regulation was further confirmed at the protein level by western blotting, which demonstrated a marked increase in GPR17 upon ZBTB7B overexpression (Figure 4A; Supplementary Figure S4B).

To explore the involvement of GPR17 in ZBTB7B-driven tumor suppression, U118 cells were transfected with GPR17-specific small interfering RNA (siRNA) si-GPR17. Successful knockdown of GPR17 was validated by western blotting (Figure 4B; Supplementary Figure S4C). Live-cell imaging demonstrated that ZBTB7B overexpression inhibited U118 cell proliferation, whereas GPR17 silencing promoted cell growth. Notably, concurrent silencing of GPR17 during ZBTB7B overexpression reversed the anticancer effect of ZBTB7B (Figure 4C). Flow cytometry demonstrated that cell apoptosis significantly increased in cells overexpressing ZBTB7B, while this effect was partially reversed by GPR17 silencing (Figure 4D and E). These results collectively suggested that ZBTB7B promoted glioma cell apoptosis through upregulation of GPR17.

It has been reported that GPR17 suppresses glioma proliferation by inducing reactive oxygen species (ROS) generation (Liu et al., 2021). To investigate the potential role of ROS in ZBTB7B-driven tumor suppression, we measured superoxide levels in U118 cells using the superoxide-sensitive dye MitoSOX Red. Immunofluorescence analysis revealed a significant increase in ROS-positive cells in the ZBTB7B-overexpression group (Figure 4F and G). Flow cytometry confirmed that intracellular MitoSOX fluorescence intensity was significantly elevated in ZBTB7B-overexpressing cells (Figure 4H). Notably, GPR17 silencing markedly attenuated ROS production (Figure 4I). Taken together, these data demonstrated that ZBTB7B exerted its inhibitory effects on glioma cell proliferation via GPR17-mediated oxidative stress induction.

Previous studies have shown that GPR17 elevates ROS by inhibiting protein kinase A (PKA) phosphorylation (Liu et al., 2021). In this study, western blot analysis revealed reduced phosphorylated PKA (p-PKA) levels in ZBTB7B-overexpressing cells. Conversely, GPR17 silencing facilitated PKA phosphorylation. Furthermore, knockdown of GPR17 rescued the ZBTB7B-induced reduction in p-PKA levels. Consistently, the abundance of the apoptotic marker cleaved Caspase3 (c-Casp3) was elevated by ZBTB7B overexpression but reduced by GPR17 silencing (Figure 4J; Supplementary Figure S4D).

### GPR17 is a downstream target of ZBTB7B in glioma cells

**Figure 5:**
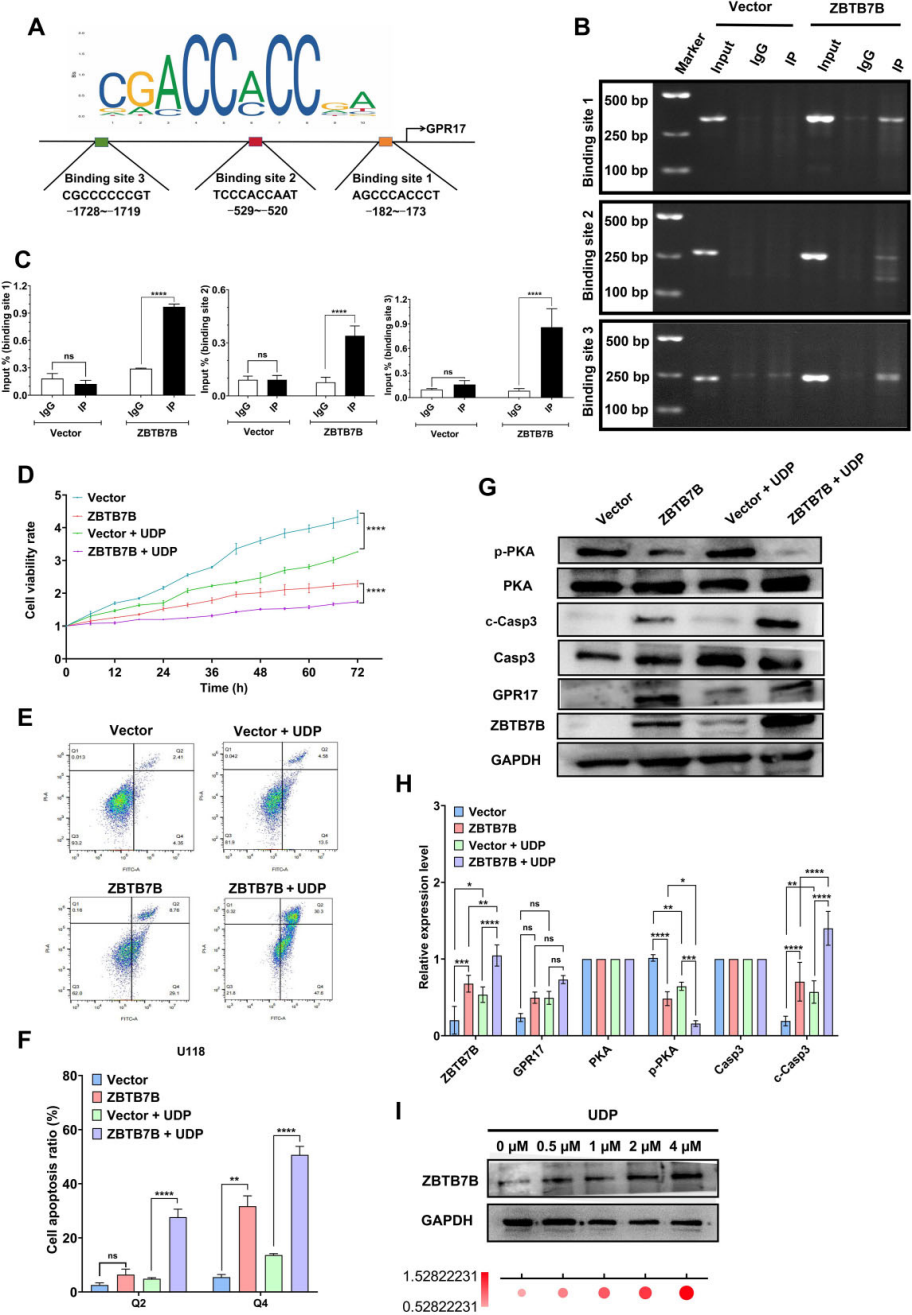
ZBTB7B specifically binds to the promoter region of the GPR17 gene, promotes its transcription, and induces cell apoptosis. (**A**) Bioinformatics analysis of the JASPAR database predicting high-affinity ZBTB7B binding sites in the GPR17 promoter. (**B** and **C**) The ChIP–PCR assay was used to analyze the sites where ZBTB7B binds to the promoter region of the GPR17 gene by agarose gel electrophoresis and quantitative analysis. (**D**–**H**) ZBTB7B-overexpressing and control U118 cells were treated with or without 1 μM UDP for 24 h. (**D**) Real-time monitoring of cell viability using a live-cell imaging system. Normalized data were statistically analyzed between groups. (**E** and **F**) Flow cytometry analysis of cell apoptosis, including early apoptosis and late apoptosis. (**G** and **H**) Western blot analysis of p-PKA, c-Casp3, ZBTB7B, and GPR17 protein expression levels, quantified with triplicate repetition. GAPDH was used as an internal reference. (**I**) Western blot analysis of ZBTB7B protein expression levels in U118 cells treated with various concentrations of UDP for 24 h. All experiments were performed independently at least three times. *P < 0.05, **P < 0.01, ***P < 0.001, ****P < 0.0001; ns, not significant.

We performed chromatin immunoprecipitation (ChIP)–PCR assays to determine whether ZBTB7B, as a transcription factor, directly binds to the GPR17 promoter to upregulate its expression. An *in silico* analysis of the JASPAR database predicted a high-confidence ZBTB7B response element containing the characteristic ‘CCRCC’ motif within the GPR17 promoter (Figure 5A). Based on this prediction, three pairs of primers were designed, targeting the putative binding sites at positions −1728/−1719, −529/−520, and − 182/−173. Subsequent ChIP–PCR assays in U118 cells confirmed that ZBTB7B directly bound to these three sites (Figure 5B and C).

GPR17, a Gi-coupled orphan receptor phylogenetically positioned between P2Y and CysLT receptors, is specifically activated by two classes of endogenous ligands to regulate downstream signaling and cellular functions. Previous studies have indicated that uridine 5′-diphosphate (UDP) serves as one of the known ligands for GPR17 and modulates diverse physiological processes (Ciana et al., 2006; Marucci et al., 2016). To elucidate the mechanism by which the ZBTB7B–GPR17 axis drives glioma cell proliferation arrest and promotes apoptosis, we evaluated the effects of UDP on cell function and downstream pathway activity in U118 cells. Our analysis revealed that UDP significantly enhanced the ZBTB7B overexpression-induced inhibition of cell proliferation (Figure 5D). Flow cytometry confirmed that the apoptotic rate significantly increased following ZBTB7B overexpression combined with UDP stimulation (Figure 5E and F). Western blot analysis further demonstrated that UDP inhibited PKA phosphorylation and upregulated c-Casp3 expression (Figure 5G and H). These results indicated that UDP, acting as a specific ligand for GPR17, participated in inhibiting survival signaling and activating apoptotic pathways, thereby reinforcing the tumor-suppressive role of the ZBTB7B–GPR17 axis in glioma cells.

Notably, UDP significantly upregulated ZBTB7B protein expression in a concentration-dependent manner (Figure 5I). Collectively, these findings demonstrated that UDP, upon binding to GPR17, initiated a positive feedback loop to enhance ZBTB7B expression, thereby potentiating the suppression of the PKA–Caspase3 signaling cascade.

### ZBTB7B promotes T cell-mediated cytotoxicity by regulating CXCL10 expression

**Figure 6:**
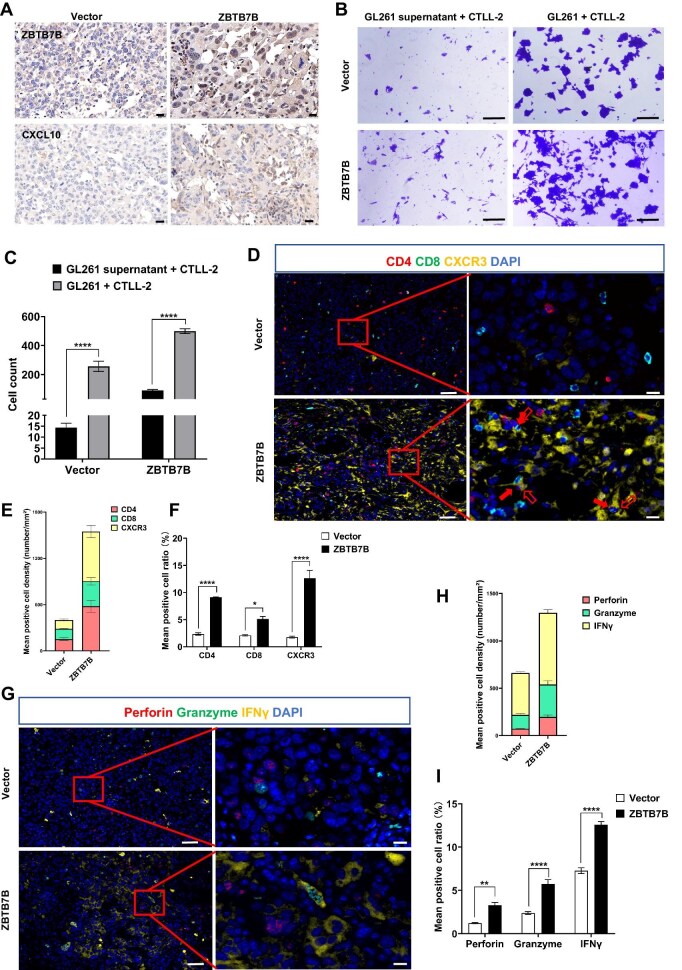
ZBTB7B promotes T cell-mediated cytotoxicity by activating CXCL10 transcription. (**A**) Immunohistochemical staining for ZBTB7B and CXCL10 in brain tumor tissues from C57BL/6 mice. Scale bar, 20 μm. (**B** and **C**) The chemotactic capacity of glioma cells (GL261) toward cytotoxic T lymphocytes (CTLL-2) was assessed using a transwell-based co-culture system. Scale bar, 50 μm. (**D**–**F**) Multiplex immunofluorescence staining of brain tumor tissues from C57BL/6 mice s for DAPI, CD4, CD8, and CXCR3 across groups. Scale bar, 50 μm. Partially enlarged view of the boxed area is shown on the right. Scale bar, 10 μm. Red arrows indicate co-localized cellular groups. (**E**) Densities of CD4^+^, CD8^+^, and CXCR3^+^ cells within tumor regions, calculated as the number of cells per square millimeter (cells/mm²). (**F**) Ratios of CD4^+^ (*P* < 0.0001), CD8^+^ (*P* = 0.01), and CXCR3^+^ (*P* < 0.0001) cells were assessed across groups using three randomly selected fields of equal size. (**G**–**I**) Multiplex immunofluorescence staining for DAPI, perforin, granzyme, and IFNγ across groups. Scale bar, 50 μm. Partially enlarged view of the boxed area is shown on the right. Scale bar, 10 μm. (**H**) Densities of perforin^+^, granzyme^+^, and IFNγ^+^ cells within tumor regions. (**I**) Ratios of perforin^+^ (*P* < 0.01), granzyme^+^ (*P* < 0.0001), and IFNγ^+^ (*P* < 0.0001) cells across groups. *P < 0.05, **P < 0.01, ****P < 0.0001.

qRT-PCR analysis revealed a significant enhancement of CXCL10 transcription following ZBTB7B overexpression (Supplementary Figure S5A). Immunohistochemical staining further confirmed substantially elevated expression of both ZBTB7B and CXCL10 in tumors from the ZBTB7B-overexpression group (Figure 6A).

Within the tumor microenvironment, CXCL10 is secreted by multiple types of immune cells, including monocytes, macrophages, and fibroblasts (Reschke et al., 2021). To assess whether ZBTB7B modulates CXCL10 levels via an exocrine mechanism, U118 cells overexpressing ZBTB7B or control vector were co-cultured with THP-1 monocytes, and CXCL10 secretion was quantified using the enzyme-linked lectin assay (ELLA) system. As shown in Supplementary Figure S5B, CXCL10 secretion increased in ZBTB7B-overexpressing cells either alone or co-culture with THP-1 monocytes. Furthermore, the differentiation of THP-1 monocytes into macrophages by phorbol 12-myristate 13-acetate treatment further elevated CXCL10 levels (Supplementary Figure S5B).

To verify the role of the ZBTB7B–CXCL10 axis in T cell recruitment, co-culture experiments were conducted using glioma cells and mouse T cells (CTLL-2). The results showed that ZBTB7B overexpression significantly increased the migration of CTLL-2 cells to the lower chamber (Figure 6B and C).

CXCL10 recruits CXCR3-expressing immune cells, including monocytes/macrophages, T cells, NK cells, and dendritic cells, to facilitate anti-tumor responses (Koper et al., 2018). Multiplex immunofluoresence staining of glioma tissues from C57BL/6 mice revealed a substantial accumulation of CXCR3^+^, CD4^+^, and CD8^+^ cells in tumor tissues overexpressing ZBTB7B (Figure 6D–F and Table 2). Notably, CXCR3/CD8 co-positive cytotoxic T cells were markedly enriched in ZBTB7B-overexpressing tumor tissue (Figure 6D, red arrows). In addition, concentrations of perforin, granzyme, and IFNγ, were significantly elevated in the ZBTB7B-overexpressing tumor tissue (Figure 6G–I and Table 3). The increase in these key effector molecules of cytotoxic immune responses further confirmed the role of ZBTB7B in promoting anti-tumor immunity.

**Table 2:** **Analysis of CD4, CD8, and CXCR3 cell positivity via multiplex immunofluorescence staining in ventricular tumor specimens from ZBTB7B-overexpressing and vector control C57BL/6 mice**.

**Parameters**	**Vector (mean ± SD)**	**ZBTB7B (mean ± SD)**
CD8^+^ T cells		
Number	81 ± 5	194 ± 29
Positivity rate (%)	2.09 ± 0.17	5.12 ± 0.84
Positive density (number/mm²)	134.72 ± 9.13	323.38 ± 48.05
CD4^+^ T cells		
Number	91 ± 11	348 ± 47
Positivity rate (%)	2.36 ± 0.37	9.12 ± 0.09
Positive density (number/mm²)	151.93 ± 18.35	579.76 ± 78.78
CXCR3^+^ cells		
Number	68 ± 9	387 ± 49
Positivity rate (%)	1.77 ± 0.30	12.64 ± 2.52
Positive density (number/mm²)	114.03 ± 14.68	645.11 ± 81.07
CD8 and CXCR3 co-localized cells		
Number	14 ± 2	148 ± 16
Positivity rate (%)	0.37 ± 0.05	3.90 ± 0.22
Positive density (number/mm²)	23.68 ± 4.01	247.37 ± 26.47
CD4 and CXCR3 co-localized cells		
Number	12 ± 2	48 ± 11
Positivity rate (%)	0.31 ± 0.05	1.27 ± 0.29
Positive density (number/mm²)	20.28 ± 2.94	80.79 ± 18.73

**Table 3:** **Analysis of perforin, granzyme, and IFNγ cell positivity via multiplex immunofluorescence staining in ventricular tumor specimens from ZBTB7B-overexpressing and vector control C57BL/6 mice**.

	**Vector**	**ZBTB7B**
**Parameters**	**(mean ± SD)**	**(mean ± SD)**
Perforin^+^ cells		
Number	44 ± 2	118 ± 12
Positivity rate (%)	1.22 ± 0.12	3.30 ± 0.56
Positive density (number/mm²)	74.00 ± 4.00	196.82 ± 20.81
Granzyme^+^ cells		
Number	87 ± 9	207 ± 23
Positivity rate (%)	2.39 ± 0.27	5.75 ± 0.85
Positive density (number/mm²)	144.93 ± 14.69	344.22 ± 38.81
IFNγ^+^ cells		
Number	265 ± 9	453 ± 19
Positivity rate (%)	7.29 ± 0.58	12.59 ± 0.59
Positive density (number/mm²)	441.63 ± 14.55	755.81 ± 31.55

These results indicated that ZBTB7B activated CXCL10 expression and promoted the infiltration of cytotoxic T lymphocytes into the tumor microenvironment. These findings suggest that this may be a key immune regulatory mechanism underlying the inhibition of glioma growth.

### Expression of ZBTB7B and GPR17 in human gliomas

The expressions of ZBTB7B and its downstream target GPR17 in human gliomas were evaluated using multiplex immunofluoresence staining on the tissue microarray, and their correlations with clinicopathological parameters were analyzed. A total of 129 glioma patients were involved (48 females and 81 males; median age 41 years; range 4–80 years), classified histopathologically as grade I (30.23%), grade II (30.23%), grade III (27.91%), and grade IV (11.63%). Tumor recurrence occurred in 68 patients (52.71%). The overall survival duration ranged 6–113 months (mean 72.73 months) (Supplementary Table S6).

**Figure 7:**
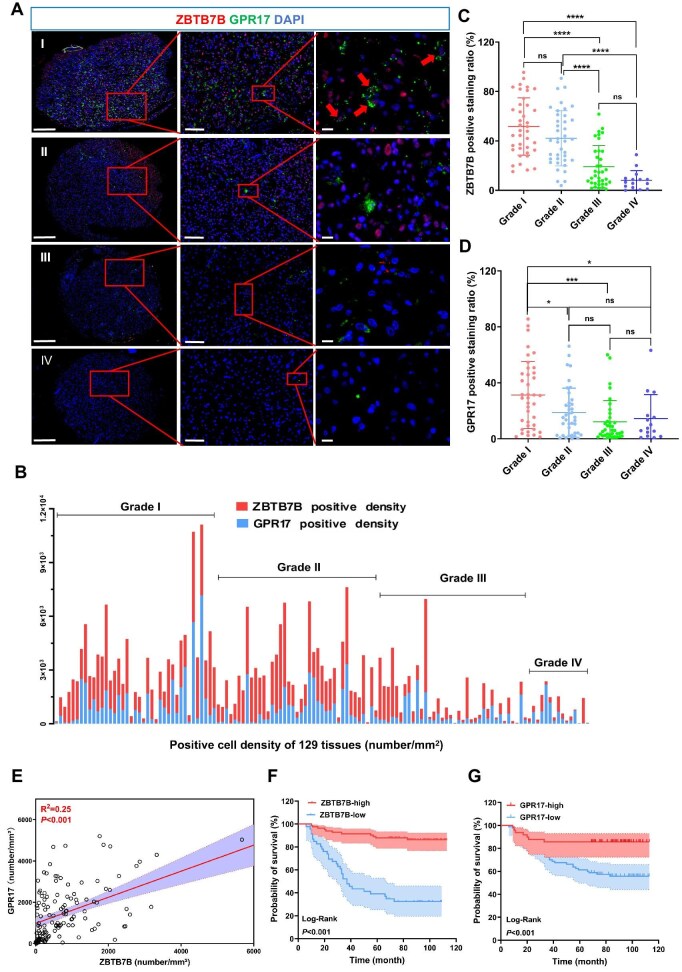
Expression levels of ZBTB7B and its target gene GPR17 are analyzed in the human glioma tissue microarray. (**A**) Multiplex immunofluorescence staining for DAPI, ZBTB7B, and GPR17 across various glioma grades. Red arrows indicate co-localized cells. Scale bar, 200 μm (left), 50 μm (middle), or 10 μm (right). (**B**) Densities of ZBTB7B^+^ and GPR17^+^ cells across glioma grades. (**C** and **D**) Ratios of ZBTB7B^+^ and GPR17^+^ cells across glioma grades. (**E**) Positive correlation between ZBTB7B and GPR17 expression levels (*R*² = 0.25, *P* < 0.001). (**F** and **G**) Kaplan–Meier survival curves illustrating overall survival in patients with high vs. low ZBTB7B or GPR17 expression (Log-Rank, *P* < 0.001). *P < 0.05, ***P < 0.001, ****P < 0.0001; ns, not significant.

Multiplex immunofluoresence analysis revealed notable variations in ZBTB7B and GPR17 expression across tumor grades (Figure 7A). ZBTB7B was highly expressed in grade I and grade II but minimal or undectable in grade IV. The expression of GPR17 exhibited a similar pattern. Cells co-expressing ZBTB7B and GPR17 were readily identified in grade I samples (Figure 7A, red arrows).

Quantitative analysis confirmed significantly different expression levels of ZBTB7B and GPR17 across grades (Figure 7B). Specifically, ZBTB7B expression was markedly higher in grade I gliomas and progressively diminished from grade II to grade IV (Figure 7C). Similarly, GPR17 exhibited strong expression in grade I, but the level declined significantly with increasing histopathological grade (Figure 7D). Pearson correlation analysis revealed a significant positive association between ZBTB7B and GPR17 expressions (*R*² = 0.25, *P* < 0.001; Figure 7E), suggesting a regulatory link between the two genes.

The associations between ZBTB7B/GPR17 expression and clinicopathological parameters are summarized in Table 4. No significant correlation was observed between ZBTB7B and gender (*P* = 0.71) or age (*P* = 0.27), whereas tumor grade was significantly inversely associated with ZBTB7B levels. Notably, 35 grade I patients exhibited high ZBTB7B expression but only 4 exhibited low expression (*P* < 0.001). Furthermore, 52 of the 61 patients without tumor recurrence exhibited high ZBTB7B levels, indicating a significant inverse correlation between elevated ZBTB7B expression and reduced tumor recurrence (*P* < 0.001). GPR17 expression presented similar correlations with clinicopathological parameters (Table 4).

**Table 4:** **Clinicopathological characteristics of glioma patients with ZBTB7B and GPR17 expression analyzed by the tissue microarray**.

		**ZBTB7B**			**GPR17**		
	**Total number**	High	Low	*P*-value	High	Low	*P*-value
Gender							
Male	81 (62.79%)	51 (62.96%)	30 (37.04%)	0.71	29 (35.80%)	52 (64.20%)	0.57
Female	48 (37.21%)	32 (66.67%)	16 (33.33%)		20 (41.67%)	28 (58.33%)	
Age (year)							
<45 years	73 (56.59%)	50 (68.49%)	23 (31.51%)	0.27	28 (38.36%)	45 (61.64%)	>0.99
≥45 years	56 (43.41%)	33 (58.93%)	23 (41.07%)		21 (37.50%)	35 (62.50%)	
Pathological grade							
I	39 (30.23%)	35 (89.74%)	4 (10.25%)	<0.001*	24 (62.54%)	15 (38.46%)	<0.001*
II	39 (30.23%)	34 (87.17%)	5 (12.82%)		15 (38.46%)	24 (62.54%)	
III	36 (27.90%)	12 (33.33%)	24 (66.67%)		7 (19.44%)	29 (80.55%)	
IV	15 (11.62%)	0 (0.00%)	15 (100%)		3 (20.00%)	12 (80.00%)	
Recurrence							
Yes	68 (52.71%)	31 (45.59%)	37 (54.41%)	<0.001*	19 (27.94%)	49 (72.06%)	0.018*
No	61 (47.29%)	52 (85.25%)	9 (14.75%)		30 (49.18%)	31 (50.82%)	

*The differences observed were statistically significant.

Kaplan–Meier survival analysis further confirmed the prognostic significance of both ZBTB7B and GPR17. Among the 83 patients with high ZBTB7B expression (63.34%), 72 survived and 11 died during the observation period, whereas of the 46 patients with low expression (35.66%), only 15 survived (Figure 7F). Similarly, elevated GPR17 expression was correlated with higher survival rates (Figure 7G).

## Discussion

Glioma is one of the most aggressive forms of brain cancer (Cahill et al., 2015), characterized by complex pathogenesis involving intricate molecular regulatory networks and dynamic interactions within the tumor immune microenvironment (Garofano et al., 2021; Bikfalvi et al., 2023). Despite significant advances in understanding the disease, effective therapeutic strategies remain elusive. This study identifies ZBTB7B as a potential molecular target for glioma treatment.

Our preliminary *in vitro* experiments revealed that ZBTB7B overexpression in glioma cells significantly reduced both cell proliferation and clonogenic potential, and the effects were mediated by the induction of apoptosis and cell cycle arrest. For further validation, *in vivo* experiments were conducted, in which U118 human glioma cells engineered to overexpress ZBTB7B were injected into the lateral ventricles of BALB/c nude mice. Remarkably, elevated ZBTB7B expression led to a substantial suppression of tumorigenicity, as evidenced by the absence of observable neoplasms in four of the treated mice. To further explore the mechanisms of tumor cell elimination, we inoculated GL261 murine glioma cells overexpressing ZBTB7B into C57BL/6 mice that possess a fully functional immune system. Consistent with the earlier findings, ZBTB7B overexpression also effectively inhibited tumorigenicity in this immune-competent model. These findings underscore the pivotal role of ZBTB7B in suppressing glioma cell growth and highlight the potential dual mechanisms by which ZBTB7B mediates tumor suppression, namely, via intrinsic cellular pathways and through interactions with the immune microenvironment.

The majority of research on ZBTB7B has focused on its role in T cell differentiation and immune regulation (Egawa, 2009; Enders et al., 2012; Cheroutre and Husain, 2013; Karimi et al., 2021), with limited efforts exploring its association with tumors. Genomic analyses of TCGA data reveal that ZBTB7B is frequently mutated or altered across a wide spectrum of human cancers. For instance, Heide et al. (2019) reported a significant positive correlation between ZBTB7B mutations and the incidence of human bladder cancer. Similarly, Zhu et al. (2024) demonstrated that decreased ZBTB7B expression in the liver promoted hepatocarcinogenesis via aberrant activation of the Akt/N-Ras signaling pathway. These findings collectively support the notion that ZBTB7B functions as a tumor suppressor gene, playing a critical role in inhibiting tumor initiation and progression across different cancer types.

To further explore the role of ZBTB7B in gliomas, we analyzed its expression in a human glioma tissue microarray and assessed its correlations with key pathological indicators. Our analysis demonstrated an inverse correlation between ZBTB7B expression and turmor severity, i.e. ZBTB7B expression is higher in lower-grade gliomas than in higher-grade gliomas. Furthermore, high levels of ZBTB7B expression are inversely correlated with tumor recurrence rates. These findings are consistent with our earlier observations and strongly suggest that ZBTB7B plays a critical role in suppressing glioma development and progression.

We further investigated the molecular mechanisms underlying the anti-cancer effects of ZBTB7B. As a transcription factor, ZBTB7B exerts functions by regulating the expression of its downstream target genes. To identify these targets, we performed RNA sequencing on U118 and GL261 cells with or without ZBTB7B overexpression. Although the ZBTB7B-regulated genes differed substantially between the two cell lines, GO and KEGG pathway enrichment analysis revealed a common enrichment in processes related to the activation of GPCR signaling, ion channel activity, T cell proliferation and migration, and cytokine–cytokine receptor interactions. Validation using a PCR array showed that ZBTB7B primarily upregulated CCL18 and GPR17 in U118 cells and IL-1β and CXCL10 in GL261 cells. The critical role of GPR17 in glioma development has been previously highlighted (Dougherty et al., 2012; Johnson et al., 2020; Mutharasu et al., 2022). In this study, a positive correlation between ZBTB7B and GPR17 expressions was observed, and both were positively associated with patient survival. This finding aligns with a previous report that GPR17 expression is significantly downregulated in human gliomas and its overexpression enhances mitochondrial ROS production by inhibiting PKA phosphorylation, ultimately inducing apoptosis (Liu et al., 2021). Another study demonstrated that the GPR17 agonist UDP-glucose inhibits the growth of primary cultured mouse glioblastomas (Dougherty et al., 2012). Our results further support that ZBTB7B overexpression disrupts glioma cell cycle progression and promotes cell apoptosis by upregulating GPR17. Knockdown of GPR17 in ZBTB7B-overexpressing cells attenuates these effects, indicating that GPR17 is critical for mediating the anti-tumor functions of ZBTB7B. Collectively, these findings suggest that ZBTB7B enhances GPR17 expression, which inhibits PKA phosphorylation, leading to increased ROS production in mitochondria that induces tumor cell apoptosis.

Recent studies have shown that the classification of the tumor microenvironment as ‘hot’ or ‘cold’ is a critical determinant of tumor progression, influencing processes such as growth, invasion, metastasis, drug resistance, and immune evasion (Galon and Bruni, 2019; Hinshaw and Shevde, 2019; Liu and Sun, 2021; Giustarini et al., 2021; Zhang et al., 2022). Advances in spatial transcriptomics and panoramic pathology have elucidated the important role of chemokines and their corresponding receptors as principal regulators of immune cell recruitment into tumor tissues. Key chemokines enriched in ‘hot’ tumors, such as interferon, CCL5, CXCL9, CXCL10, and CXCL16, are produced by antigen-presenting cells (e.g. dendritic cells and macrophages) as well as by tumor cells themselves (House et al., 2020; Reschke et al., 2021; Reschke and Gajewski, 2022; Hoch et al., 2022; Bill et al., 2023). Among these, CXCL10 plays a pivotal role in recruiting CXCR3-positive CD8^+^ cytotoxic T cells to the tumor site (Korniejewska et al., 2011; Fenwick et al., 2015; Reynders et al., 2019; Karin, 2020; Chen et al., 2024). A recent study demonstrated the co-localization of CXCL9 and CXCL10 with LAG3^+^ T cells expressing CCL4 or CXCL13, which facilitates the formation of a thermophilic tumor microenvironment (Hoch et al., 2022). Similarly, another study showed that mice with CXCL10 knockout fail to recruit CXCR3-positive CD8^+^ T cells to tumors, highlighting the importance of CXCL10 in anti-tumor immunity (House et al., 2020). In alignment with these findings, our study demonstrated that ZBTB7B induces CXCL10 expression in glioma cells and monocytes, suggesting its potential role in modulating the tumor immune microenvironment to suppress tumorigenicity. Using ELLA and immunohistochemistry, we confirmed that ZBTB7B upregulates CXCL10 and promotes the recruitment of CXCR3-positive CD8^+^ T cells to the tumor microenvironment. Through multiplex immunofluorescence analysis, we further revealed significantly elevated levels of perforin, granzyme, and IFNγ in tumors with high ZBTB7B levels, verifying the role of ZBTB7B in promoting CXCL10-mediated immune responses in gliomas. These findings corroborate previous research demonstrating the anti-tumor functions of ZBTB7B. This is exemplified by the work of Chen et al. (2022) who demonstrated that ZBTB7B inhibits the ERK signaling pathway by inducing STPG1, thereby facilitating T cell activation and suppressing gastric cancer progression. Similarly, Xia et al. (2021) reported that ZBTB7B activates the NF-κB pathway through inactivation of TNFRSF12A, resulting in enhanced T cell proliferation. Taken together, these findings support the hypothesis that elevated ZBTB7B expression fosters a immunologically ‘hot’ tumor microenvironment characterized by robust immune activation, which impedes tumor progression.

**Figure 8:**
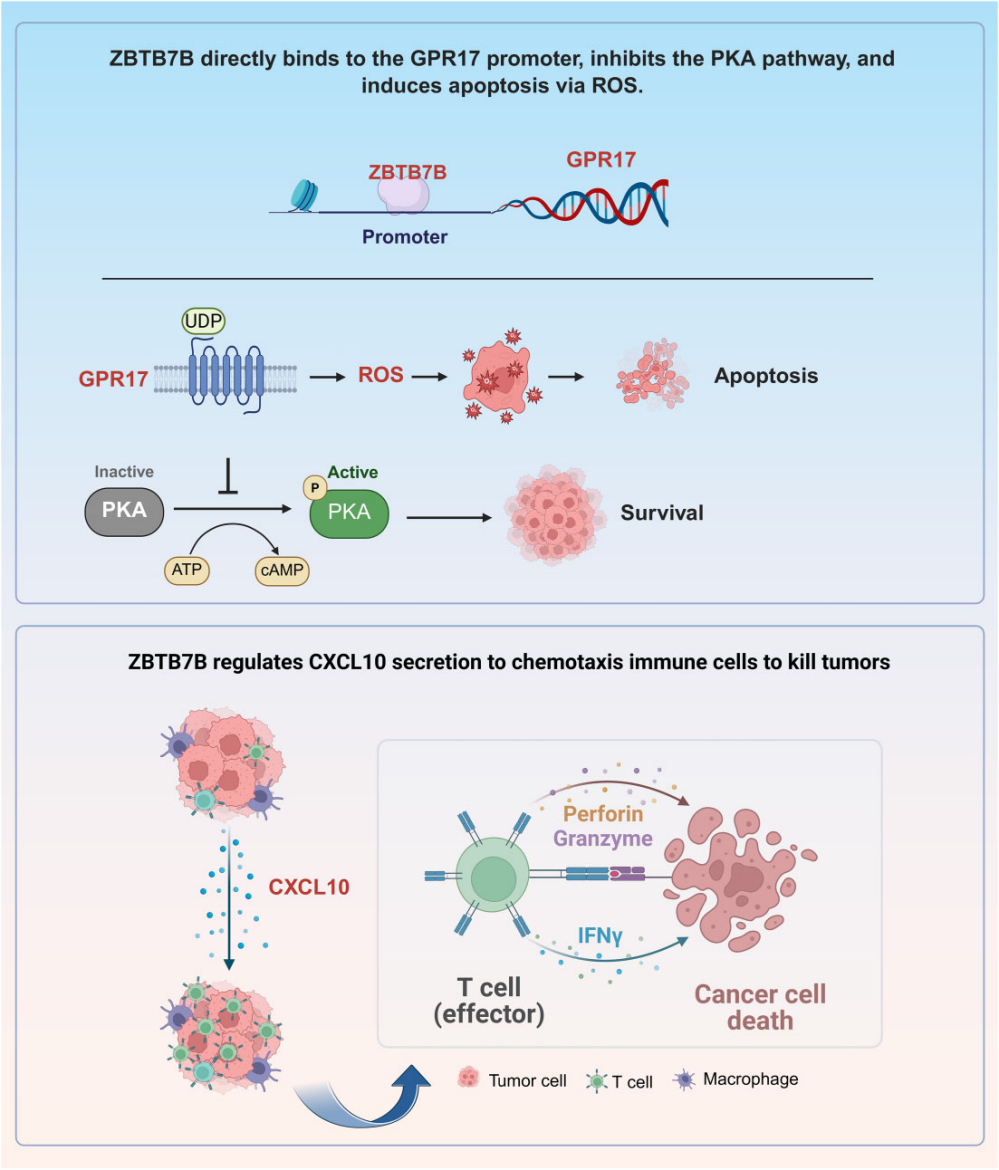
Schematic illustration of the mechanism by which ZBTB7B inhibits glioma progression.

As delineated in Figure 8, ZBTB7B acts as a transcriptional activator through direct promoter binding and activation of GPR17. Enhanced GPR17 expression suppresses PKA phosphorylation, thereby elevating mitochondrial ROS production and inducing glioma cell apoptosis. Concurrently, ZBTB7B-driven upregulation of CXCL10 promotes infiltration of CD4^+^ and CD8^+^ T cells into the tumor microenvironment, intensifying immune-mediated cytotoxicity via effector molecules such as perforin, granzyme, and IFNγ. These dual pathways—mitochondria-driven apoptotic signaling and immune-mediated cytotoxicity—highlight the multifaceted role of ZBTB7B in suppressing glioma tumorigenicity and progression. This study advances the understanding of the molecular and immunological mechanisms underlying ZBTB7B-mediated glioma inhibition and lays the foundation for developing novel therapeutic approaches.

Furthermore, we found that UDP significantly upregulated ZBTB7B protein expression. The finding demonstrates that UDP establishes a positive feedback loop to enhance ZBTB7B expression and synergistically modulates the PKA–Caspase3 signaling cascade, thereby potentiating the tumor-suppressive effects of the ZBTB7B–GPR17 axis. Our work not only reveals the UDP–GPR17–ZBTB7B axis as a novel therapeutic target for glioma but also highlights its potential for clinical translation in the precision therapy of gliomas.

## Materials and methods

### Cell lines

The human glioma cell line U118MG (RRID: CVCL_0633) and mouse glioma cell line GL261 (RRID: CVCL_Y003) were cultured in Dulbecco’s Modified Eagle’s Medium (Gibco) supplemented with 10% fetal bovine serum (FBS; DiNing) and 1% penicillin–streptomycin (100 U/ml penicillin and 100 μg/ml streptomycin sulfate). The IL-2-dependent cytotoxic T lymphocyte line CTLL-2 (RRID: CVCL_0227) was maintained in IL-2-supplemented complete medium (RPMI-1640 medium with 10% FBS and 100 U/ml recombinant murine IL-2). Cells were maintained at 37°C in a humidified incubator with 5% CO_2_, authenticated by short tandem repeat profiling, and confirmed free of mycoplasma contamination.

### Cell transfection

The human ZBTB7B coding sequence (GenBank Gene ID: BC012070) was cloned into GV492 (Ubi-MCS-3FLAG-CBh-gcGFP-IRES-puromycin) or GV341 (Ubi-MCS-3FLAG-SV40-puromycin) lentiviral vectors (GeneChem). Lentiviruses expressing ZBTB7B or control vector were generated by GeneChem. Glioma cells infected with these lentiviruses were selected with 0.5 mg/ml puromycin (Sigma–Aldrich). ZBTB7B overexpression was verified by qRT-PCR and western blotting.

For intracranial glioma modeling, cells overexpressing ZBTB7B or control vector were co-transfected with pLenti-CBh-3×FLAG-Luc2-tCMV-mNeonGreen-F2A-Puro-WPRE (ObiO Cat# H7656) and selected with puromycin to establish stable lines.

siRNAs targeting GPR17 and the corresponding negative control were obtained from Genepharma, and sequences are listed in Supplementary Table S1. siRNA transfections were performed using jetPRIME transfection reagent (Polyplus Cat# 114-15) according to the manufacturer’s instructions.

### Intracranial injection of tumor cells into mice

Animal experiments were approved by the Animal Use Committee at Xi’an Jiaotong University. Five-week-old male C57BL/6 (RRID: IMSR_JAX:000664) and BALB/c nude mice (RRID: MGI:5650033), purchased from Xi’an Jiaotong University Animal Experiment Center, were housed under pathogen-free conditions. Mice were randomly divided into control and ZBTB7B-overexpression groups (*n* = 5 per group). All analyses were performed in a blinded manner.

Intracranial glioma xenografts were established by injecting U118 or GL261 cells (ZBTB7B-overexpressing or control) into mouse lateral ventricles as previously described (McCutcheon et al., 2000). Tumor cell suspensions (10000 cells/μl, total volume 5 μl) were injected 3 mm deep, with the needle retracted by 1 mm prior to injection at 1 μl/min. Tumor growth was monitored using the Maestro Automated In Vivo Imaging System (Xenogen Caliper).

### ChIP assay

ChIP assays for GPR17 were performed in U118 cells with or without ZBTB7B overexpression. Approximately 5 × 10^6^ cells were fixed with 1% formaldehyde at room temperature for 10 min, and cross-linking was quenched by adding 0.125 M glycine. Chromatin was sheared into fragments of 200–500 bp using a sonicator. Soluble chromatin was incubated overnight at 4°C with rotation with 10 μg of anti-ZBTB7B antibody (RRID: AB_2217245) and 5 μg of control anti-IgG antibody (RRID: AB_3672282). Protein A/G magnetic beads (Beyotime Cat# P2080S) were then added, followed by incubation with rotation for 4 h at 4°C. The beads were sequentially washed with low-salt buffer, high-salt buffer, LiCl buffer, and TE buffer. Chromatin complexes were eluted by adding 500 μl of elution buffer and heating at 65°C for 6 h to reverse cross-links. DNA was purified using a commercial kit (Beyotime Cat# D0033) and subjected to agarose gel electrophoresis to confirm fragment sizes of 200–500 bp. Enriched sequence of the GPR17 promoter region was quantified by qPCR using specific primers (Supplementary Table S2).

### Ethics approval statement

The animal experiment involved in this research was reviewed and approved by the Biomedical Ethics Committee of Health Science Center of Xi’an Jiaotong University. The human tissue microarray study involved in this research was reviewed and approved by the Ethics Committee of Shanghai Outdo Biotech Company.

### Statistical analysis

All *in vitro* experiments were independently performed at least three times. Data are presented as mean ± standard error of the mean, if not specified. Group differences were analyzed using two-tailed unpaired Student’s *t*-test (normally distributed data) or two-way analysis of variance followed by multiple comparisons. Associations between biomarkers and clinical parameters were assessed by chi-square or Fisher’s exact tests. Survival analyses were performed using the Kaplan–Meier method. Image quantification was conducted with ImageJ (RRID: SCR_003070) and Aipathwell software. Statistical analyses and graphs were generated with GraphPad Prism 10 (RRID: SCR_002798). A *P*-value <0.05 was considered statistically significant.

## Supplementary Material

mjaf043_Supplemental_File

## Notes

**Author contributions:** Y.C.: writing—review & editing and project administration; L.Z.: conceptualization, investigation, and writing—original draft; H.Z., C.W., A.J., F.Z., S.Y., and X.Y.: methodology, investigation, validation, and formal analysis; J.R., H.L., and C.T.: resources and investigation; X.W.: Software. All authors conducted a thorough critical review of the manuscript regarding its intellectual content, verified their complete access to all data pertaining to the study, and accepted responsibility for the submission of the manuscript for publication.
