# Supplementary material for: ZBTB7B inhibits glioma tumorigenicity by upregulating GPR17 and CXCL10
Source: J Mol Cell Biol. 2025 Nov 26;17(11):mjaf043. doi: 10.1093/jmcb/mjaf043 (PMC13127139; doi:10.1093/jmcb/mjaf043)
Supplement: mjaf043_Supplemental_File [file mjaf043_supplemental_file.pdf]

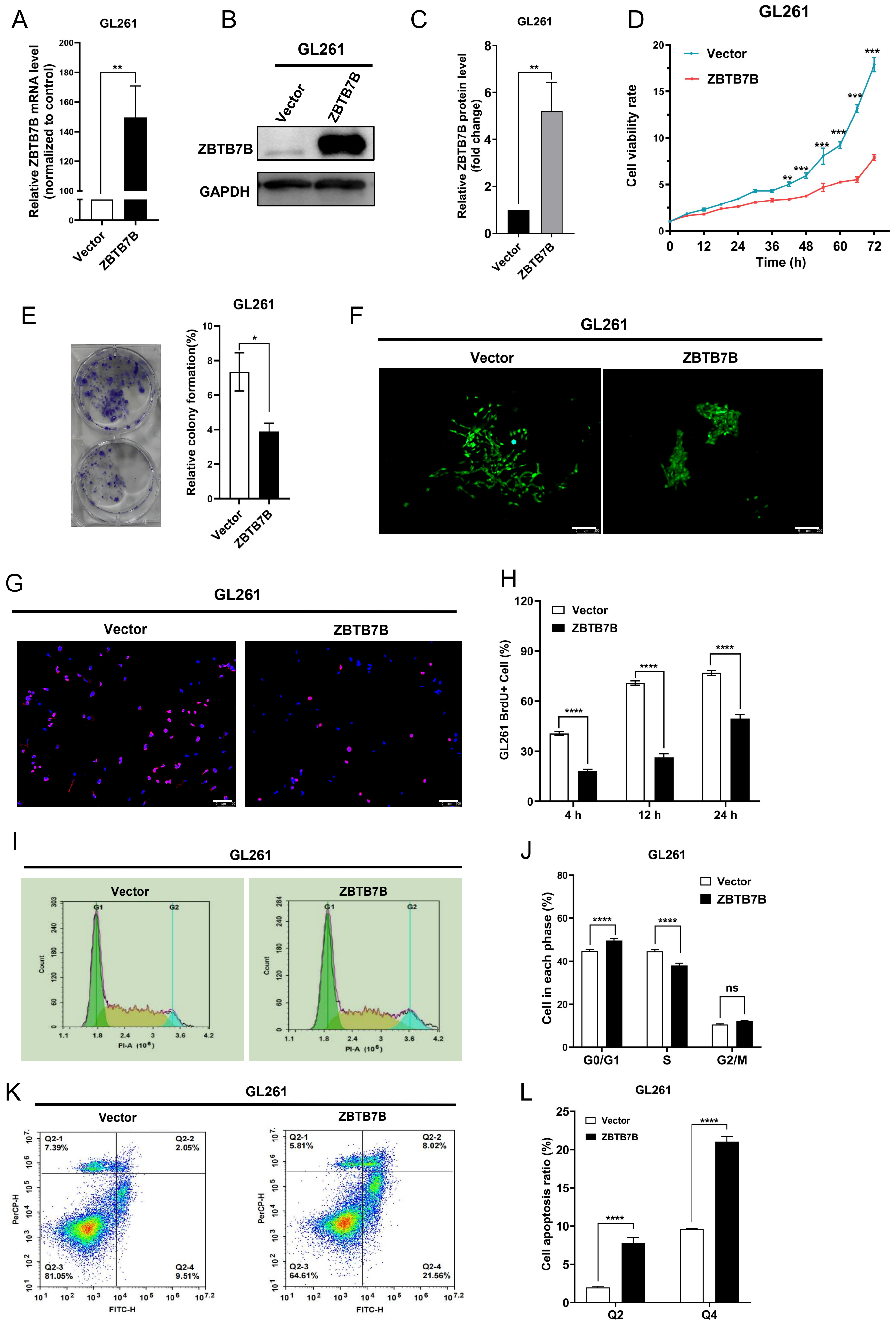

A
GL261
Relative ZBTB7B mRNA level (normalized to control)
Vector
ZBTB7B
B
GL261
ZBTB7B
GAPDH
C
GL261
Relative ZBTB7B protein level (fold change)
D
GL261
Vector
ZBTB7B
Cell viability rate
Time (h)
E
GL261
Relative colony formation(%)
F
GL261
Vector
ZBTB7B
G
GL261
H
GL261 BrdU+ Cell (%)
4 h
12 h
24 h
I
GL261
Count
PI-A (10⁶)
J
GL261
Cell in each phase (%)
G0/G1
S
G2/M
ns
K
GL261
PerCP-H
FITC-H
Q2-1 7.39%
Q2-2 2.05%
Q2-3 81.05%
Q2-4 9.51%
Q2-1 5.81%
Q2-2 8.02%
Q2-3 64.61%
Q2-4 21.56%
L
GL261
Cell apoptosis ratio (%)
Q2
Q4

Supplementary Figure S1

ZBTB7B inhibits the growth and survival in GL261 cells

(A) Quantitative real-time PCR (qRT-PCR) analysis of ZBTB7B mRNA expression in GL261 cells after stable ZBTB7B overexpression.

(B) Western blotting analysis of ZBTB7B protein expression in GL261 cells after stable ZBTB7B overexpression.

(C) Quantification (n=3 biologically independent experiments) of ZBTB7B protein expression in GL261 cells after stable ZBTB7B overexpression.

(D) Real-time monitoring of cell viability by using a live-cell imaging system. Data were normalized and statistically analyzed to compare viability between ZBTB7B-overexpressing and control in GL261 cells.

(E) Two-dimensional (2D) clonogenic assays evaluating the effect of ZBTB7B on GL261 cells clonogenic potential. Quantitative analysis of the clonogenic formation rate (%) is shown.

(F) Three-dimensional (3D) clonogenic assays demonstrating the effect of ZBTB7B on GL261 cells growth in 3D culture. Scale bar: 250 μm.

(G) Immunofluorescence staining of BrdU-labeled GL261 cells to quantify proliferation. Scale bar: 100 μm.

(H) Quantitative analysis of BrdU-positive cells as a proportion of the total cell population in GL261 cells.

(I) Flow cytometry analysis of cell cycle alterations in GL261 cells after stable ZBTB7B overexpression.

(J) Quantitative assessment of cell cycle distribution in GL261 cells after ZBTB7B overexpression.

(K) Flow cytometry analysis of apoptosis rates in GL261 cells after ZBTB7B overexpression.

(L) Quantitative analysis of apoptosis rates (%) in GL261 cells after ZBTB7B overexpression.

All experiments were conducted independently three times.

Statistical significance: *$p < 0.05$; **$p < 0.01$; ***$p < 0.001$; ****$p < 0.0001$.

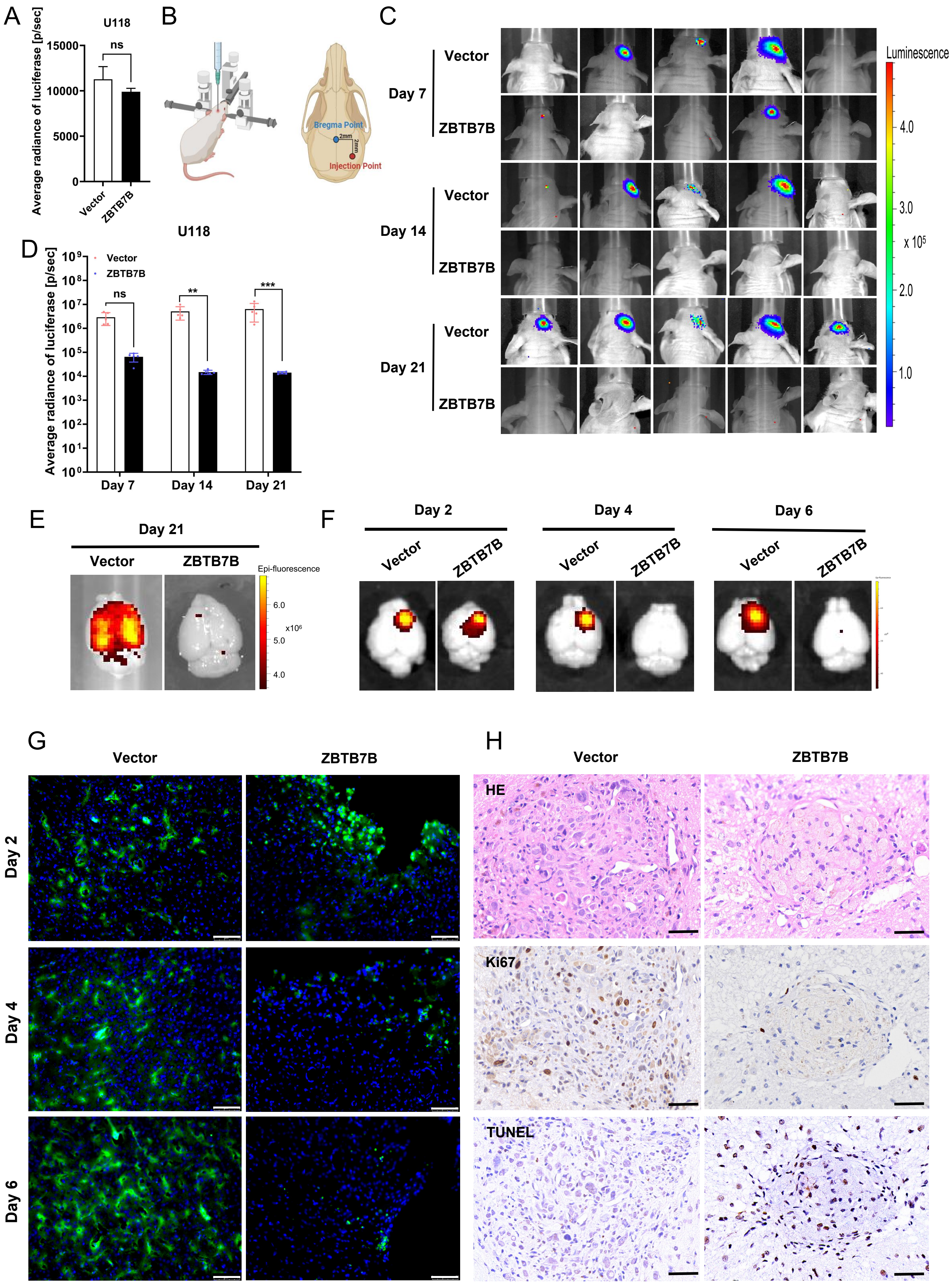

A
U118
ns
Average radiance of luciferase [p/sec]
15000
10000
5000
0
Vector
ZBTB7B
B
Bregma Point
2mm
2mm
Injection Point
C
Day 7
Vector
ZBTB7B
Day 14
Vector
ZBTB7B
Day 21
Vector
ZBTB7B
Luminescence
4.0
3.0
x 10^5
2.0
1.0
D
U118
Vector
ZBTB7B
ns
**
***
Average radiance of luciferase [p/sec]
Day 7
Day 14
Day 21
E
Day 21
Vector
ZBTB7B
Epi-fluorescence
6.0
x10^6
5.0
4.0
F
Day 2
Day 4
Day 6
Vector
ZBTB7B
G
Vector
ZBTB7B
Day 2
Day 4
Day 6
H
Vector
ZBTB7B
HE
Ki67
TUNEL

Supplementary Figure S2

ZBTB7B inhibits tumorigenicity of human glioma cells in BALB/c nude mice.

(A) Evaluation of luciferase fluorescence intensity in U118 cells cultured *in vitro.*

(B) The xenograft tumor model in the lateral ventricle of BALB/c nude mice.

(C) Tumor growth in brain tissue was monitored on days 7, 14, and 21 using the IncuCyte® Small Animal *in vivo* Imaging System. Vector (n = 5); ZBTB7B (n = 5).

(D) Quantitative analysis of luciferase fluorescence intensity *in vivo.*

(E) GFP fluorescence signals of tumor cells detected in brain tissue collected 21 days post-implantation.

(F) GFP fluorescence intensity of tumor cells in brain tissue measured on days 2, 4, and 6.

(G) Fluorescence microscopy of frozen brain tissue sections, GFP signals of tumor cells staining with DAPI. Scale bar: 75 µm.

(H) The tumor tissues were stained with HE, Ki67, and TUNEL. Scale bar: 50 µm.

Statistical significance: $^{**}p < 0.01$; $^{***}p < 0.001$; ns = not significant.

A

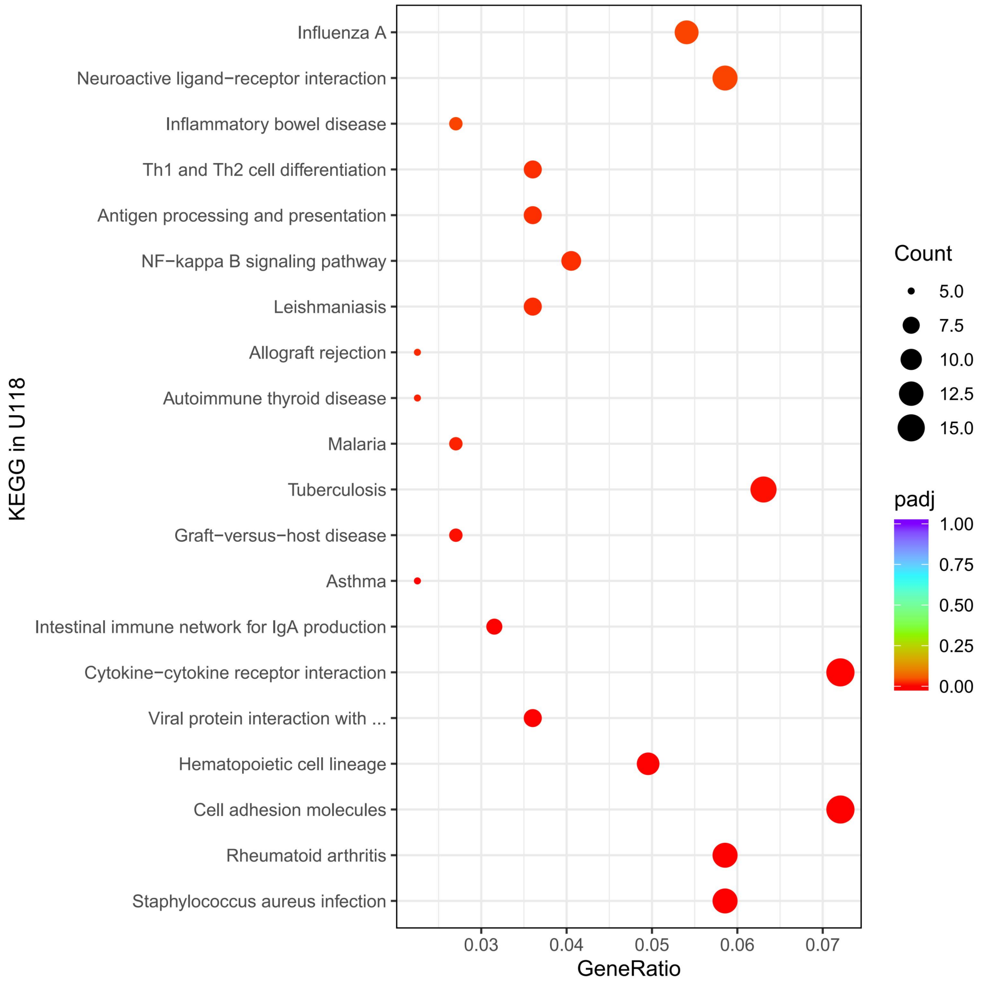
Influenza A
Neuroactive ligand−receptor interaction
Inflammatory bowel disease
Th1 and Th2 cell differentiation
Antigen processing and presentation
NF−kappa B signaling pathway
Leishmaniasis
Allograft rejection
Autoimmune thyroid disease
Malaria
Tuberculosis
Graft−versus−host disease
Asthma
Intestinal immune network for IgA production
Cytokine−cytokine receptor interaction
Viral protein interaction with ...
Hematopoietic cell lineage
Cell adhesion molecules
Rheumatoid arthritis
Staphylococcus aureus infection
KEGG in U118
0.03
0.04
0.05
0.06
0.07
GeneRatio
Count
5.0
7.5
10.0
12.5
15.0
padj
1.00
0.75
0.50
0.25
0.00

B

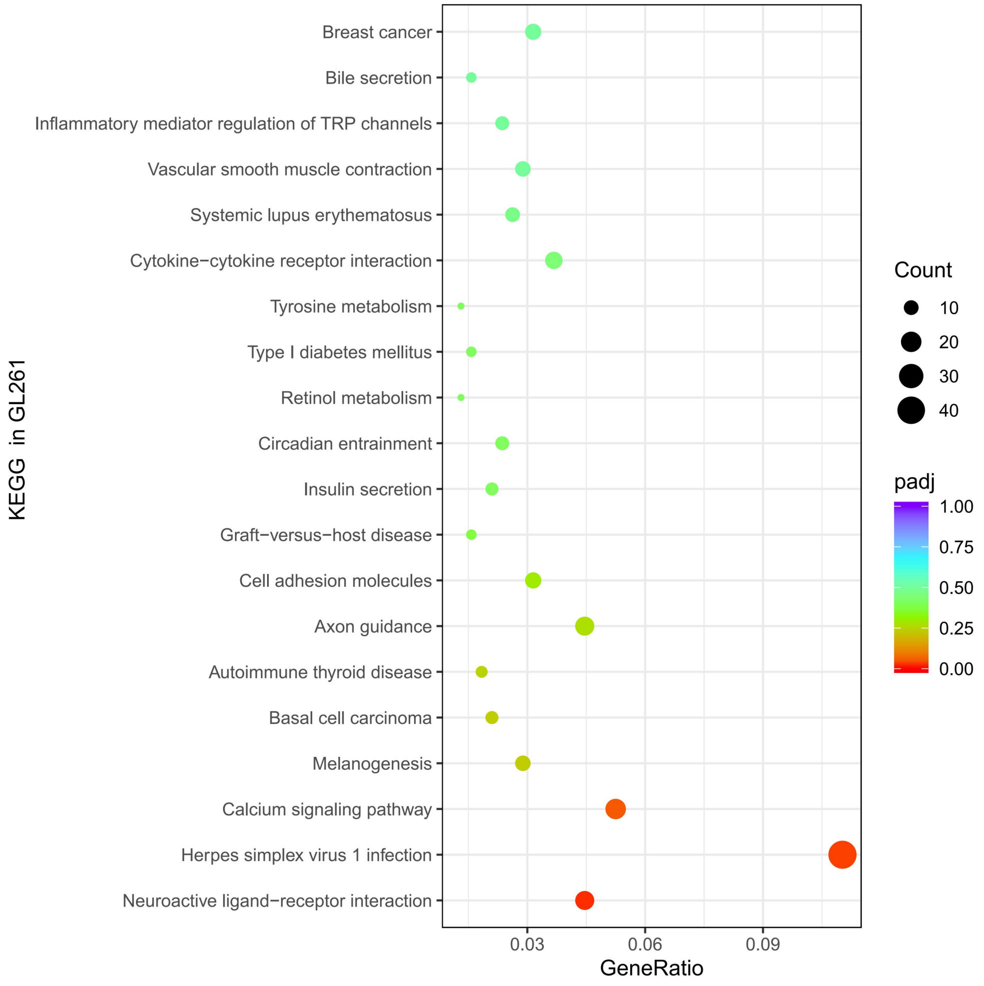
Breast cancer
Bile secretion
Inflammatory mediator regulation of TRP channels
Vascular smooth muscle contraction
Systemic lupus erythematosus
Cytokine−cytokine receptor interaction
Tyrosine metabolism
Type I diabetes mellitus
Retinol metabolism
Circadian entrainment
Insulin secretion
Graft−versus−host disease
Cell adhesion molecules
Axon guidance
Autoimmune thyroid disease
Basal cell carcinoma
Melanogenesis
Calcium signaling pathway
Herpes simplex virus 1 infection
Neuroactive ligand−receptor interaction
KEGG in GL261
0.03
0.06
0.09
GeneRatio
Count
10
20
30
40
padj
1.00
0.75
0.50
0.25
0.00

Supplementary Figure S3

KEGG pathway enrichment analysis

The U118 (A) and GL261 (B) cells overexpress ZBTB7B, KEGG pathway enrichment analysis was performed on the upregulated differentially expressed genes (DEGs).

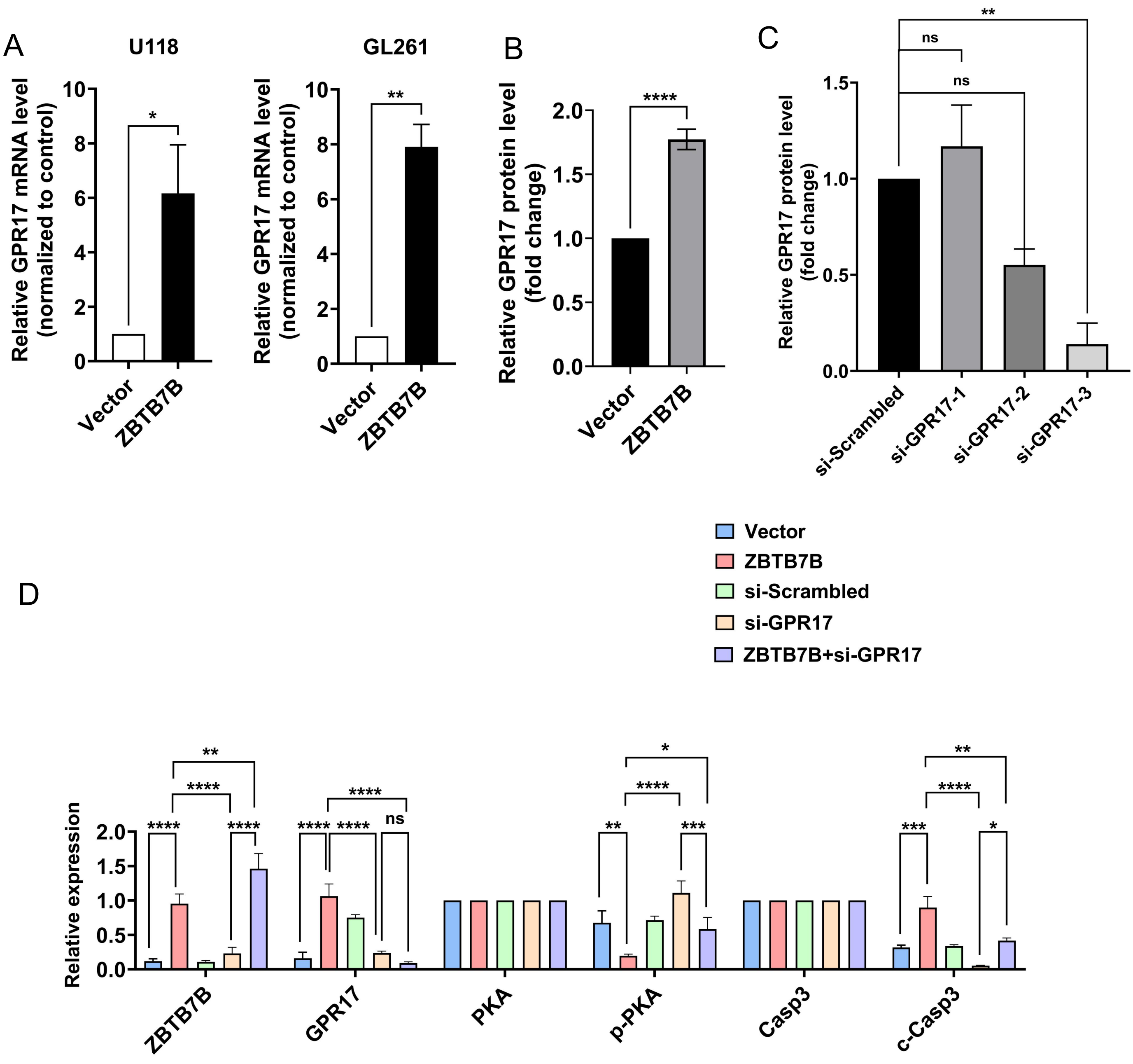
A
U118
GL261
Relative GPR17 mRNA level (normalized to control)
Vector
ZBTB7B
B
Relative GPR17 protein level (fold change)
C
si-Scrambled
si-GPR17-1
si-GPR17-2
si-GPR17-3
ns
D
Vector
ZBTB7B
si-Scrambled
si-GPR17
ZBTB7B+si-GPR17
Relative expression
ZBTB7B
GPR17
PKA
p-PKA
Casp3
c-Casp3

Supplementary Figure S4

Related to Figure 4

(A) Quantitative real-time PCR (qRT-PCR) analysis of GPR17 mRNA level in U118 and GL261 cells after stable ZBTB7B overexpression.

(B) Quantification (n=3 biologically independent experiments) of GPR17 level in U118 cells with and without ZBTB7B overexpression as determined by Western blotting (Related to Figure 4A).

(C) Quantification (n=3 biologically independent experiments) of effect of three small interfering RNAs (siRNAs) on inhibiting endogenous GPR17 protein expression by Western blotting (Related to Figure 4B).

(D) Quantitative analysis of levels of phosphorylated protein kinase A (p-PKA), cleaved Caspase-3, ZBTB7B, and GPR17 by Western blotting (Related to Figure 4J).

Statistical significance: *$p < 0.05$; **$p < 0.01$; ***$p < 0.001$; ****$p < 0.0001$; ns = not significant.

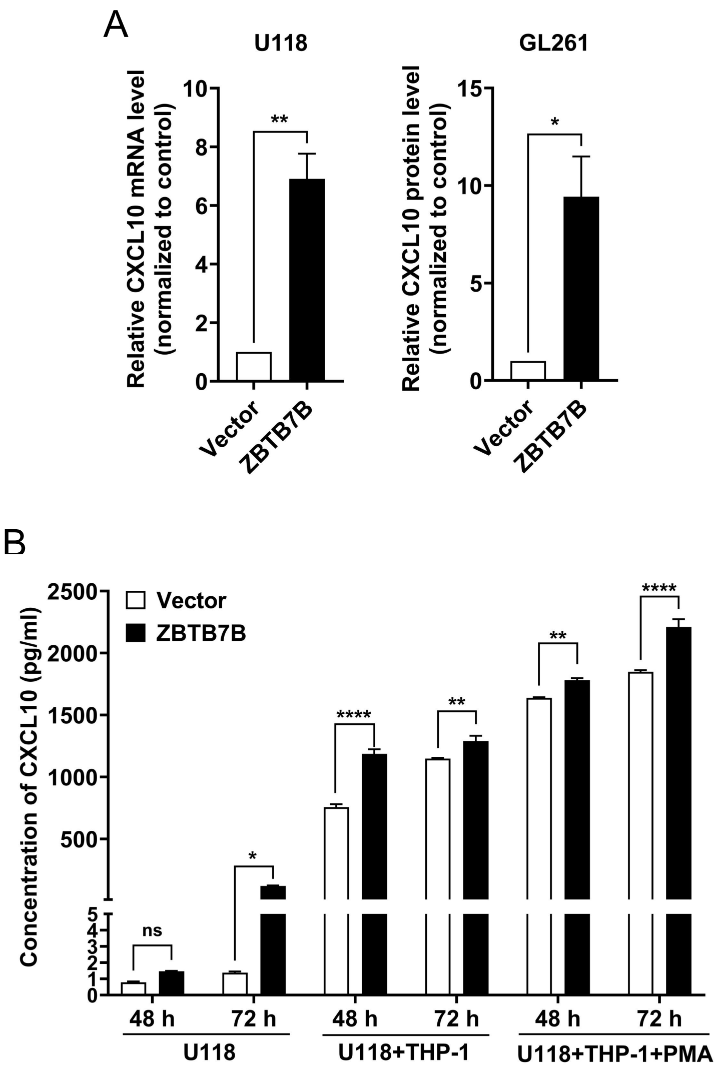
A
U118
GL261
Relative CXCL10 mRNA level (normalized to control)
Relative CXCL10 protein level (normalized to control)
**
*
Vector
ZBTB7B
B
Vector
ZBTB7B
Concentration of CXCL10 (pg/ml)
ns
*
****
**
**
****
48 h
72 h
U118
U118+THP-1
U118+THP-1+PMA

Supplementary Figure S5

Related to Figure 6

(A) Quantitative real-time PCR (qRT-PCR) analysis of CXCL10 mRNA expression in U118 and GL261 cells after stable ZBTB7B overexpression.

(B) Quantification of CXCL10 levels across induced differentiation groups using an ultra-sensitive, fully automated microfluidic Ella system.

Statistical significance: *$p < 0.05$; **$p < 0.01$; ***$p < 0.001$; ****$p < 0.0001$; ns = not significant.

Supplementary Table S1

The list of siRNA sequences designed to target GPR17

	Sense 5'-3'	Antisense 5'-3'
si-Scrambled	UUCUCCGAACGUGUCACGUTT	ACGUGACACGUUCGGAGAATT
si-GPR17-1	GCAUGAAUGGCCUUGAAGUTT	ACUUCAAGGCCAUUCAUGCTT
si-GPR17-2	CAGGUCUGAUCACCAACUUTT	AAGUUGGUGAUCAGACCUGTT
si-GPR17-3	GCAUGAUCGCCAUAGUGCUTT	AGCACUAUGGCGAUCAUGCTT

Supplementary Table S2
The list of primers and their detailed sequences

Gene name	Primer name	Primer sequence (5'->3')
ZBTB7B	Forward primer	GCTAGTGTTTGGAGAGAGGGC
	Reverse primer	TCCCCATCTTCTCCACGTCTC
GAPDH	Forward primer	GACATCAAGAAGGTGGTGAAGC
	Reverse primer	GCTGTTGAAGTCAGAGGAGACC
GPR17	Forward primer	AGCAGCTAGAGGATGTCCAAAC
	Reverse primer	TTTGGCTGGAGTCAGAGCCT
gpr17-Mouse	Forward primer	GGAGCACCATCTAGAGCACCCT
	Reverse primer	GGCTGCCTCCAGACCGTTCAT
CXCL10	Forward primer	GTGGCATTCAAGGAGTACCTC
	Reverse primer	GCCTTCGATTCTGGATTCAGACA
cxcl10-Mouse	Forward primer	CCCACGTGTTGAGATCATTG
	Reverse primer	CAGTTAAGGAGCCCTTTTAGACC
Gapdh-Mouse	Forward primer	CAGAACATCATCCCTGCATC
	Reverse primer	CTGCTTCACCACCTTCTTGA
GPR17-ChIP Binding Site 1	Forward primer	TCAGGGTCAGCTGACCGAAT
	Reverse primer	GCCTACATGCTGAGTCTGG
GPR17-ChIP Binding Site 2	Forward primer	CATGACCCCACTCCACATTC
	Reverse primer	CGGCTGTCTGGAAGAACTG
GPR17-ChIP Binding Site 3	Forward primer	CATCCCCTTCTGAAACGTCC
	Reverse primer	GAGGGGTCCCTTGTTGTGTC
GAPDH-ChIP	Forward primer	TACTAGCGGTTTTACGGGCG
	Reverse primer	TCGAACAGGAGGAGCAGAGAGCGA

Supplementary Table S3

The list of cytokine and chemokine targets of Murine evaluated through PCR arrays (wc-mRNA0038-M)

mouse	1	2	3	4	5	6	7	8	9	10	11	12
A	Adipoq	Ccl17	Ccl5	Csf3	Cxcl14	Hc	Il15	Il2	Il4	Mapk14	Tgfb2	Vegfa
B	Bmp2	Ccl19	Ccl7	Ctf1	Cxcl16	Ifna2	Il16	Il21	Il5	Mif	Thpo	Xcl1
C	Bmp4	Ccl2	Cd14	Cx3cl1	Cxcl3	Ifng	Il17a	Il22	Il6	Mstn	Tnf	Il1rn
D	Bmp6	Ccl20	Cd40lg	Cxcl1	Cxcl5	Il10	Il17f	Il23a	Il7	Nodal	Tnfrsf11b	Il33
E	Bmp7	Ccl22	Cd70	Cxcl10	Cxcl9	Il11	Il18	Il24	Il9	Osm	Tnfsf10	Ltb
F	Ccl1	Ccl24	Cntf	Cxcl11	Epo	Il12a	Il1a	Il27	Lif	Pf4	Tnfsf11	Spp1
G	Ccl11	Ccl3	Csf1	Cxcl12	Fasl	Il12b	Il1b	Il3	Lta	Ppbp	Tnfsf13b	Trem1
H	Ccl12	Ccl4	Csf2	Cxcl13	Gpi1	Il13	Actb	Gapdh	Hprt1	B2m	NTC	NTC

Supplementary Table S4

The list of chemokines and receptors targets of human evaluated through PCR arrays (wc-mRNA0033-H)

human	1	2	3	4	5	6	7	8	9	10	11	12
A	ACKR2	CCL14	CCL21	CCL3	CCR3	CD40	CX3CR1	CXCL2	CXCR4	IL11RA	IL16	IL8
B	ACKR3	CCL15	CCL22	CCL4	CCR4	CKLF	CXCL1	CXCL3	CXCR5	XCL1	IL1B	IL9R
C	ACKR4	CCL16	CCL23	CCL5	CCR5	CMKLR1	CXCL10	CXCL5	CXCR6	IL12RB1	IL4	CXCL16
D	C5	CCL17	CCL24	CCL7	CCR6	CMTM1	CXCL11	CXCL6	DARC	IL12RB2	IL4R	CXCR3
E	C5AR1	CCL18	CCL25	CCL8	CCR7	CMTM2	CXCL12	CXCL9	FPR1	IL13RA1	IL5RA	IL10RB
F	CCL1	CCL19	CCL26	CCR1	CCR8	CMTM3	CXCL13	CXCR1	GPR17	IL13RA2	IL6R	IL15RB
G	CCL11	CCL2	CCL27	CCR10	CCR9	CMTM4	CXCL14	CXCR2	IL10RA	IL15RA	IL6ST	IL7R
H	CCL13	CCL20	CCL28	CCR2	CCRL2	CX3CL1	ACTB	GAPDH	HPRT1	18S	NTC	NTC

Supplementary Table S5

The list of antibodies with detailed specifications

primary antibody	Dilution	KDa	Host	Appication	Antibody Brands	Cat#	RRID
ZBTB7B	1:1000	75	Rabbit	WB	Cell signaling	#13205	AB_2798147
GPR17	1:750	41	Rabbit	WB	Abcam	ab316105	AB_3662934
PKA	1:1000	41	Rabbit	WB	Gene Tex	GTX104934	AB_2037751
p-PKA	1:1000	42	Rabbit	WB	Cell signaling	#5661	AB_10707163
Cleaved-caspase3	1:750	17	Rabbit	WB	Gene Tex	GTX110543	AB_10722709
Caspase3	1:750	32	Rabbit	WB	Gene Tex	GTX110543	AB_10722709
GAPDH	1:5000	36	Mouse	WB	Proteintech	HRP-60004	AB_2737588
P21	1:200	21	Rabbit	WES	Abcam	ab109520	AB_10860537
P27	1:200	27	Rabbit	WES	Abcam	ab32034	AB_2244732
P18	1:200	19	Rabbit	WES	Abcam	ab31543	AB_2114473
P53	1:200	53	Mouse	WES	Abcam	ab26	AB_303198
p-P53	1:200	53	Rabbit	WES	Abcam	ab33889	AB_776988
CDK2	1:200	33-34	Rabbit	WES	Abcam	ab32147	AB_726775
CDK4	1:200	34	Rabbit	WES	Abcam	ab108357	AB_10867218
CDK6	1:200	37	Rabbit	WES	Abcam	ab124821	AB_10999714
GAPDH	1:1000	36	Mouse	WES	Proteintech	HRP-60004	AB_2737588
ZBTB7B	1:200		Rabbit	mIF	Proteintech	11341-1-AP	AB_2217245
GPR17	1:200		Rabbit	mIF	Affinity Biosciences	DF4966	AB_2837319
CD4	1:1000		Rabbit	mIF	Servicebio	GB15064	AB_3095557
CD8	1:1000		Mouse	mIF	Servicebio	GB12068	AB_2905512
CXCR3	1:200		Rabbit	mIF	Affinity Biosciences	DF7113	AB_2839067
Perforins	1:200		Rabbit	mIF	Thermo Fisher Scientific	PA5-109315	AB_2854726
Granzymes	1:200		Rabbit	mIF	Proteintech	13588-1-AP	AB_2114429

INF-γ	1:500	Rabbit	mIF	Proteintech	15365-1-AP	AB_2123037
CXCL10	1:200	Mouse	IHC/ELLA	Thermo Fisher Scientific	MA5-23774	AB_2609319
Ki67	1:500	Rabbit	IHC	Proteintech	27309-1-AP	AB_2756525
BrdU	1:200	Mouse	IF	Proteintech	66241-1-lg	AB_2881630
ZBTB7B	10μg	Rabbit	IP	Proteintech	11341-1-AP	AB_2217245
IgG	5μg	Rabbit	IP	Proteintech	98136-1-RR	AB_3672282

**secondary antibody**	**Dilution**	**Appication**	**Ex/Em (nm)**	**Antibody Brands**	**Cat#**	**RRID**
HRP conjugated Goat Anti-Rabbit IgG (H+L)	1:10000	WB		Servicebio	GB23303	AB_2811189
HRP conjugated Goat Anti-Mouse IgG (H+L)	1:10000	WB		Servicebio	GB23301	AB_2904020
HRP conjugated Goat Anti-Rabbit IgG (H+L)	1∶500	IHC/mIF		Servicebio	GB23303	AB_2811189
HRP conjugated Goat Anti-Mouse IgG (H+L)	1∶500	IHC/mIF		Servicebio	GB23301	AB_2904020
HRP conjugated Goat Anti-Rabbit IgG (H+L)		WES		Servicebio	GB23303	AB_2811189
HRP conjugated Goat Anti-Mouse IgG (H+L)		WES		Servicebio	GB23301	AB_2904020
Alexa Fluor® 594-conjugated Goat Anti-Mouse IgG (H+L)	1∶200	IF	591/614	Thermo Fisher Scientific	A-11005	AB_2534073

**Tyramide signal amplification (TSA)**	**Dilution**	**Ex/Em (nm)**	**Appication**	**Antibody Brands**	**Cat#**
iF488-Tyramide	1:500	491/516	mIF	Servicebio	G1231
iF546-Tyramide	1:500	541/557	mIF	Servicebio	G1251
iF440-Tyramide	1:500	434/480	mIF	Servicebio	G1250
iF647-Tyramide	1:500	656/670	mIF	Servicebio	G1232
DAPI	1:500	359/457	mIF	Servicebio	G1012

Supplementary Table S6

Clinical information from tissue microarrays of 129 glioma patients

Patient No.	State of survival	Overall survival (Month)	Gender	Age	Grade#	Recurrence
1	Survive	113	Female	37	II	No
2	Survive	113	Female	20	I	No
3	Survive	113	Male	44	I-II	No
4	Survive	112	Female	63	III	Yes
5	Survive	112	Male	42	I-II	No
6	Deaths	22	Male	53	IV	Yes
7	Survive	111	Female	11	I	No
8	Survive	109	Male	17	II-III	No
9	Survive	109	Female	47	II	No
10	Survive	108	Female	34	I	No
11	Survive	108	Female	59	II	No
12	Survive	108	Male	80	II	Yes
13	Survive	108	Female	28	I-II	No
14	Survive	106	Male	62	II-III	No
15	Deaths	83	Male	66	II	Yes
16	Survive	106	Female	55	I-II	No
17	Survive	106	Female	46	I-II	No
18	Survive	106	Female	30	II	Yes
19	Survive	105	Female	49	II	Yes
20	Survive	105	Male	61	II	No
21	Survive	105	Male	44	II	Yes
22	Survive	104	Male	39	II-III	Yes
23	Survive	104	Male	56	II	No
24	Survive	103	Male	12	I	No
25	Survive	103	Male	66	I-II	No
26	Survive	102	Male	6	I-II	No
27	Survive	102	Male	5	I	No
28	Survive	101	Female	43	II-III	Yes
29	Survive	101	Male	32	I	No
30	Survive	101	Male	57	II	No
31	Deaths	67	Female	55	III	Yes
32	Survive	101	Female	61	I	No
33	Survive	100	Male	34	II	No
34	Survive	100	Male	44	II	Yes
35	Deaths	33	Male	37	IV	Yes
36	Survive	99	Male	20	I-II	No
37	Deaths	66	Female	41	III	Yes
38	Survive	99	Male	41	II	No
39	Survive	99	Male	68	II-III	Yes
40	Survive	98	Male	31	II	Yes
41	Deaths	59	Male	63	II	Yes
42	Survive	98	Male	65	I-II	Yes
43	Deaths	50	Female	57	II-III	Yes
44	Survive	97	Male	57	II	Yes
45	Survive	97	Female	6	I	No

46	Survive	97	Male	47	I-II	Yes
47	Survive	97	Female	42	I-II	No
48	Survive	97	Male	60	I-II	Yes
49	Survive	96	Male	63	II	Yes
50	Survive	96	Male	20	I-II	No
51	Deaths	57	Male	69	II	Yes
52	Survive	96	Female	42	II	Yes
53	Survive	95	Female	26	I	No
54	Deaths	30	Female	41	III	Yes
55	Survive	94	Female	36	II	No
56	Deaths	11	Male	22	IV	Yes
57	Survive	94	Male	20	II-III	Yes
58	Survive	93	Male	36	I-II	No
59	Survive	93	Female	18	I-II	No
60	Survive	93	Female	42	II-III	No
61	Deaths	55	Male	41	II	Yes
62	Deaths	73	Male	58	II-III	Yes
63	Survive	92	Male	35	II-III	No
64	Deaths	15	Female	43	IV	Yes
65	Deaths	37	Male	46	II-III	Yes
66	Survive	91	Female	17	I-II	No
67	Survive	91	Male	33	II-III	No
68	Survive	89	Male	4	II	No
69	Deaths	33	Male	79	I-II	Yes
70	Deaths	20	Male	74	II	Yes
71	Survive	88	Male	50	I-II	No
72	Survive	88	Male	32	II	Yes
73	Survive	88	Male	36	III	Yes
74	Survive	88	Male	38	I	No
75	Survive	87	Female	18	I	No
76	Survive	87	Female	42	II	Yes
77	Deaths	55	Female	45	II-III	Yes
78	Survive	87	Male	20	II-III	No
79	Deaths	10	Male	60	IV	Yes
80	Deaths	33	Female	33	III	Yes
81	Survive	86	Male	37	I-II	No
82	Deaths	17	Female	60	IV	Yes
83	Survive	85	Female	35	I-II	No
84	Survive	85	Male	36	II	No
85	Survive	85	Female	49	I	No
86	Survive	84	Male	19	II	No
87	Deaths	11	Male	59	III	Yes
88	Survive	84	Male	41	I-II	No
89	Deaths	32	Male	56	IV	Yes
90	Survive	82	Male	33	III	No
91	Survive	81	Female	38	I	No
92	Deaths	41	Male	46	IV	Yes
93	Deaths	34	Female	58	II-III	Yes
94	Deaths	23	Female	30	III	Yes
95	Deaths	34	Male	65	IV	Yes
96	Deaths	12	Male	41	IV	Yes

97	Deaths	26	Male	49	III	Yes
98	Deaths	6	Male	78	IV	Yes
99	Survive	77	Female	48	II	Yes
100	Survive	77	Male	40	I-II	No
101	Survive	76	Male	41	II	Yes
102	Survive	76	Female	45	II	No
103	Survive	75	Male	48	II	No
104	Deaths	18	Male	30	III	Yes
105	Deaths	23	Male	52	II-III	Yes
106	Survive	75	Female	26	II-III	Yes
107	Deaths	10	Male	62	IV	Yes
108	Survive	74	Male	56	II	No
109	Survive	74	Male	15	II	No
110	Deaths	19	Male	67	III	Yes
111	Survive	72	Male	30	I	No
112	Deaths	29	Female	70	II-III	Yes
113	Survive	72	Female	36	II-III	Yes
114	Deaths	26	Male	41	II	Yes
115	Deaths	9	Male	63	IV	Yes
116	Deaths	10	Male	45	IV	Yes
117	Survive	71	Male	37	II	No
118	Deaths	11	Female	69	III	Yes
119	Survive	71	Female	19	II	No
120	Survive	71	Male	49	II	No
121	Survive	71	Female	66	I	No
122	Deaths	14	Male	41	IV	Yes
123	Deaths	21	Male	56	III	Yes
124	Survive	70	Female	20	I	No
125	Survive	70	Female	28	II	Yes
126	Deaths	37	Male	57	III	Yes
127	Survive	69	Male	10	I	No
128	Survive	69	Female	36	II-III	Yes
129	Deaths	39	Male	40	II-III	Yes

#

Grades I and I-II are classified as Grade I;

Grade II includes Grade II only;

Grades II-III and III are classified as Grade III;

Grade IV remains as Grade IV.

Supplementary Materials and methods

Quantitative Real-Time Polymerase Chain Reaction (qRT-PCR)

Total RNA was extracted using TRIzol reagent (jetPRIME Cat# 19201ES60) according to the manufacturer's protocol. RNA concentration and purity were assessed using a NanoDrop spectrophotometer (Thermo Fisher Scientific USA). Complementary DNA (cDNA) was synthesized using gDNA Digester Plus Reagent (Yeasen Cat# 11120ES60). Quantitative real-time PCR (qRT-PCR) was performed using an iQ5 Multicolor qRT-PCR Detection System (Bio-Rad USA) and Hieff qPCR SYBR Green Master Mix (Yeasen Cat# 11201ES08). GAPDH served as an internal control for normalization. Relative mRNA expression levels were calculated using the $2^{-\Delta\Delta Ct}$ method. Primer sequences are provided in Supplementary Table S2.

PCR Array

U118 and GL261 cells stably overexpressing ZBTB7B were analyzed using PCR arrays (WCGENE PCR Array Plate Wcgene Biotech Shanghai China). Murine cytokine and chemokine panels (Cat# wc-mRNA0038-M) and human chemokine and receptor panels (Cat# wc-mRNA0033-H) were utilized. Specific genes analyzed are listed in Supplementary Tables S3 and S4. Data were analyzed using software provided by Wcgene Biotech. Genes exhibiting $|\log2FoldChange| \geq 1.0$ were considered biologically significant.

Western Blotting

Proteins were extracted using RIPA buffer (MCE Cat# HY-K1001), and 10–20 µg of protein lysates were separated on a 10% SDS-PAGE gel (Servicebio Cat# G2046). Proteins were then transferred onto PVDF membranes (Millipore Cat# IPFL00005). The membranes were blocked with a Protein-Free Rapid Closure Solution (Servicebio Cat# G2052) and incubated overnight at 4°C with primary

antibodies. HRP-conjugated secondary antibodies were detected using ECL-enhanced chemiluminescence (Servicebio Cat# G2161). The antibodies and their corresponding RRIDs are listed in Supplementary Table S5.

Digital Western (WES)

Protein lysates (0.2 mg/mL) from U118 cells overexpressing ZBTB7B or control vector were prepared using the Sample Preparation Kit (Protein Simple Cat# PS-TP09). Automated capillary electrophoresis and protein detection were performed using the WES system (Protein Simple USA). Protein samples were mixed with 0.1× sample buffer and 5× master fluorescent solution, denatured at 95°C for 5 min, and loaded along with a biotinylated ladder, primary antibodies (1:100 dilution), HRP-conjugated secondary antibodies, chemiluminescent substrates, and wash buffers into assay plates for analysis by the WES system. Antibodies and their RRIDs are detailed in Supplementary Table S5.

Ella

Cells were plated in 12-well plates and co-cultured with THP-1 cells (PrIcella RRID: CVCL_0006) in specialized medium (PrIcella Cat# CM-0233). Following induction with PMA (Yeasen Cat# 50601ES03) at a concentration of 100 μg/mL for 48 and 72 hours, the supernatants were collected. Chemokine CXCL10 levels were quantified using the Ultra-Sensitive Fully Automated Microfluidic ELISA System (Protein Simple USA) according to the manufacturer's instructions.

Cell Viability Assay

Cell viability was assessed by seeding cells in 96-well plates at densities of 3000-5000 cells per well. Following treatment, cell viability was monitored using the CBM Fully Automated Live Cell Imaging Analysis System (BioTek USA) at 6-hour intervals for 72 hours.

Colony Formation Assay and 3D Colony Imaging

Cells were plated in 6-well plates at densities of 1000, 3000 and 5000 cells per well and incubated at 37°C with 5% CO_2 for 10-14 days. Colonies were fixed with paraformaldehyde and stained with 0.1% crystal violet. For 3D colony imaging, the Matrigel Basement Membrane Matrix (Corning Cat# 354253) was diluted with pre-cooled DMEM. Subsequently, 1.5 mL of the Matrigel dilution was added to each well of a 6-well plate. Images were captured using a fluorescence microscope (Zeiss Germany).

Cell Cycle and Apoptosis Analysis

Cells were harvested by trypsinization, washed twice with phosphate-buffered saline (PBS), and fixed overnight at 4°C in 70% ice-cold ethanol. After fixation, cells were washed twice with PBS, treated with RNase A (0.1 mg/mL) and propidium iodide (PI 0.05 mg/mL Beyotime Cat# C1052-50T) for 15 min at room temperature, and analyzed for cell cycle distribution. Apoptosis was assessed using an Annexin V-FITC/PI Apoptosis Detection Kit (Yeasen Cat# 40302ES50) according to the manufacturer's protocol. Cell cycle and apoptosis were evaluated by flow cytometry (FACS Calibur BD Biosciences USA).

BrdU Immunofluorescence Assay

Cells were seeded into 8-well plates (5000 cells/well) and incubated overnight for adherence. Subsequently, cells were treated with BrdU (20 µg/mL Sigma Cat# 19-160) for 4 h. At 4, 12, and 24 h post-incubation, cells were washed with PBS, fixed with 4% paraformaldehyde, permeabilized with 2 M HCl (37 °C), and neutralized with 0.1 M sodium tetraborate. Following a 30-min blocking step, cells were incubated overnight at 4°C with anti-BrdU primary antibody (RRID: AB_2881630), then incubated for 2 h with secondary antibody. Nuclei were counterstained with 1 µM DAPI for 20 min. Antibodies and their RRIDs are provided in Supplementary Table S5.

Mitochondrial Superoxide (MitoSOX) Assay

Mitochondrial reactive oxygen species (ROS) were measured using MitoSOX Red (Thermo Fisher Cat# M36008) at a final concentration of 5 µM. Cells were incubated with the dye for 1 hour at room temperature, following the manufacturer's instructions. Fluorescence intensity and the number of positively stained cells were analyzed using flow cytometry and fluorescence microscopy.

Hematoxylin and Eosin (H&E) Staining

Paraffin-embedded sections (5 µm) were deparaffinized, rehydrated, and stained with hematoxylin (Servicebio Cat# G1005-1) for 8 min. After differentiation in 1% hydrochloric acid-ethanol solution, sections were rinsed, treated with 0.6% ammonia water, and counterstained with eosin (Servicebio Cat# G1005-2) for 2 min. Slides were dehydrated, cleared with graded alcohols and xylene, mounted with neutral gum, and analyzed microscopically.

Immunohistochemistry (IHC)

Sections underwent deparaffinization, rehydration, and antigen retrieval. Endogenous peroxidase was quenched with 3% hydrogen peroxide, and nonspecific binding was blocked with serum. Primary antibodies were incubated overnight at 4°C, followed by secondary antibody incubation at room temperature. Signal visualization was performed with DAB chromogen, and nuclei were counterstained using hematoxylin. Sections were dehydrated, cleared, mounted, and examined via Nikon Eclipse C1 microscope and scanned using a 3DHISTECH Pannoramic MIDI system.

TUNEL Assay

TUNEL staining employed the DAB (SA-HRP) TUNEL Cell Apoptosis Detection Kit on deparaffinized tissue sections. Samples were treated with recombinant TdT enzyme and biotin-dUTP

labeling mix, followed by streptavidin-HRP. DAB served as chromogen, and hematoxylin was applied for nuclear counterstaining. Slides were sealed with neutral gum after staining.

Tissue Microarray and Multiplex Immunofluorescence (mIF)

The Brain Glioma Group Microarray (HBraG177Su01) containing 129 tumor samples and associated clinicopathologic data were acquired from Shanghai Outdo Biotech Co., Ltd. (http://www.superchip.com.cn/index.html). Clinical details are provided in Supplementary Table S6. Following deparaffinization and antigen retrieval, sections were blocked and sequentially incubated with primary antibodies and HRP-labeled secondary antibodies (RRID: AB_2811189; Supplementary Table S5). Signals were amplified using Tyramide signal amplification (TSA), nuclei counterstained with DAPI, and autofluorescence minimized using quenchers (Servicebio Cat# G1221 and G1401). Fluorescence imaging was performed with a Zeiss Axioscan 7 scanner, and positive cell ratios and densities were quantified using Aipathwell software.

Evaluation of mIF Staining

Scanned sections were analyzed using Aipathwell® software to calculate the positive cell ratio and positive cell density. Signal-positive tumor cells were quantified as a proportion of total tumor cells and categorized as follows:

- Greater than 50% (+++): High expression
- Between 20% and 50% (++): Moderate expression
- Between 5% and 20% (+): Low expression
- Less than 5% (-): Minimal or no expression

Cases classified as "+++" or "++" were considered to have high expression of the target proteins, while those categorized as "+" or "-" were classified as having low expression.

RNA Sequencing (RNA-Seq)

RNA sequencing was performed by Novogene (Beijing China). RNA integrity was assessed using the RNA Nano 6000 Assay Kit on an Agilent Bioanalyzer 2100 system (Agilent Technologies USA). Sample clustering utilized the TruSeq PE Cluster Kit v3-cBot-HS (Illumina). Differential gene expression was analyzed using DESeq2 (RRID: SCR_015687). Gene Ontology (RRID: SCR_002811) and KEGG pathway enrichment analyses (RRID: SCR_012773) were conducted using clusterProfiler (RRID: SCR_016884). Genes with adjusted p-value ≤ 0.05 and $|\log2FoldChange| \geq 1.0$ were defined as differentially expressed. RNA-seq data were deposited publicly (GSE290306).

Transwell Migration Assay

GL261 cells with or without ZBTB7B overexpression were plated in the lower chamber at a density of 1×10^6 cells/mL in DMEM medium supplemented with 10% FBS. CTLL-2 cells were resuspended in serum-free medium and added to the upper chamber at a density of 5×10^6 cells/mL. The cells were co-cultured at 37 °C under 5% CO_2 for 24 hours. After co-culture, non-migrated cells on the upper surface of the membrane were removed using cotton swabs. CTLL-2 cells that had migrated to the underside of the membrane were fixed with 4% paraformaldehyde for 15 minutes and then stained with 0.1% crystal violet for 20 minutes. Images were captured using an inverted light microscope. For statistical analysis, cells in four randomly selected fields per membrane were counted.

Statistical Analysis

All in vitro experiments were independently performed at least three times. Data are presented as mean ± SEM. Group differences were analyzed using two-tailed unpaired Student's t-test (normally distributed data) or two-way ANOVA followed by multiple comparisons. Associations between biomarkers and clinical parameters were assessed by chi-square or Fisher's exact tests. Survival

analyses were performed using the Kaplan-Meier method. Image quantification was conducted with ImageJ (RRID: SCR_003070) and Aipathwell software. Statistical analyses and graphs were generated with GraphPad Prism 10 (RRID: SCR_002798). A p-value < 0.05 was considered statistically significant.
